# A total-evidence phylogenetic approach to understanding the evolution, depth transitions, and body-shape changes in the anglerfishes and allies (Acanthuriformes: Lophioidei)

**DOI:** 10.1371/journal.pone.0322369

**Published:** 2025-05-02

**Authors:** Alex J. Maile, W. Leo Smith, Matthew P. Davis

**Affiliations:** 1 Biodiversity Institute and Department of Ecology and Evolutionary Biology, University of Kansas, Lawrence, Kansas, United States of America; 2 Department of Biology & Chemistry, St. Cloud State University, St. Cloud, Minnesota, United States of America; Laboratoire de Biologie du Développement de Villefranche-sur-Mer, FRANCE

## Abstract

The anglerfishes and allies (Lophioidei) are a diverse group of fishes with over 400 carnivorous species that are renowned for their remarkable hunting behavior employing a modified first dorsal-fin spine to lure prey and adaptations such as “pseudo-walking,” bioluminescence, and parasitic sexual dimorphism. Gaining a comprehensive understanding of their evolutionary history has been challenging, as previous studies using DNA sequence data or morphological traits have provided either inconsistent or contradictory results. We present a new comprehensive phylogenetic framework for the evolution of the Lophioidei, combining ultraconserved elements (UCEs), mitochondrial DNA sequence data, and morphological characters. Our findings reveal a monophyletic Lophioidei, positioned as the sister group to the Tetraodontoidei within a broader acanthuriform radiation. Goosefishes (Lophioideo) emerge as the stem anglerfish lineage, forming a sister clade with frogfishes (Antennarioideo) + batfishes (Ogcocephaloideo) and coffinfishes (Chaunacoideo) + deep-sea anglerfishes (Ceratioideo). We expanded the Antennariidae to include all previous frogfish (antennarioid) families as subfamilies while proposing a new subfamily, Fowlerichthyinae, to produce a stable monophyletic taxonomy for the Antennarioideo. Further, we evaluated previously and newly proposed morphological characters to diagnose the Lophioidei and Lophioideo. Our investigations demonstrated that several traditional synapomorphies are no longer diagnostic for the Lophioidei. Based on our phylogeny, a geometric morphometric analysis revealed significant differences in body shape among lophioid infraorders, especially in frogfishes and deep-sea anglerfishes, indicating the importance of habitat transitions on body-shape evolution. This study, integrating genome-scale nuclear, mitochondrial, and morphological data, provides a total-evidence perspective on the evolutionary history of lophioids and sheds light on their specializations and body-shape changes as they transitioned across and within environments.

## Introduction

**Fig 1:**
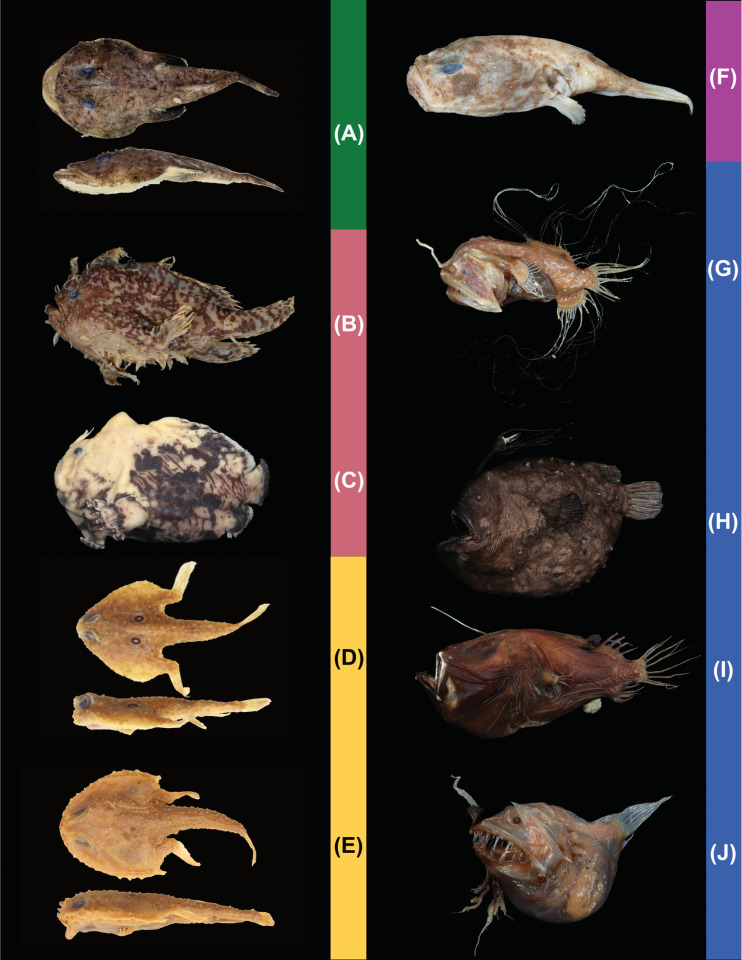
Images of representative anglerfishes (Lophioidei). Background colors associated with lophioid infraorders. Lophioideo: (A) *Lophiomus setigerus*, 160 mm SL, FMNH 121120 (dorsal and lateral view; anterior to left). Antennarioideo: (B) *Histrio histrio*, 138 mm SL, LACM 8975-1 (lateral view; anterior to left). (C) *Antennatus strigatus*, 81 mm SL, LACM 20677 (lateral view; anterior to left). Ogcocephaloideo: (D) *Zalieutes elater*, 75 mm SL, FMNH 89523 (dorsal and lateral view; anterior to left). (E) *Dibranchus atlanticus*, 137 mm SL, FMNH 65256 (dorsal and lateral view; anterior to left). Chaunacoideo: (F) *Chaunax* sp., 158 mm SL, LACM 44750-3 (lateral view; anterior to left). Ceratioideo: (G) *Caulophryne polynema*, 131 mm SL, LACM 33923-1 (lateral view; anterior to left). (H) *Himantolophus sagamius*, 348 mm SL, LACM 60082-1 (lateral view; anterior to left). (I) *Cryptopsaras couseii*, 175 mm SL, LACM 11231-1 (lateral view; anterior to left). (J) *Linophryne densiramus*, 60 mm SL, LACM 38440-1 (lateral view; anterior to left).

The anglerfishes and their allies (Lophioidei herein; Lophiiformes in [1]) are a fascinating lineage of percomorph fishes that have captivated the imagination of the public and scientists. One of their first accounts dates to *Historia Animalia* that included the description of some of the anatomical and physiological habits of the “fishing-frog” that drew Aristotle himself to the extraordinary behavior of the anglerfishes’ luring behavior. The Lophioidei consists of 408 living species classified in 74 genera and 21 families [2]. These include (Fig 1) the goosefishes and monkfishes (Lophioideo herein; Lophioidei of [1]); 30 species [2], frogfishes (Antennarioideo herein; Antennarioidei of [1]); 69 species [2], batfishes (Ogcocephaloideo herein; Ogcocephaloidei of [1]); 98 species [2], coffinfishes (Chaunacoideo herein; Chaunacoidei of [1]); 33 species [2], and deep-sea anglerfishes (Ceratioideo herein; Ceratioidei of [1]); 178 species [2].

### Interrelationships of Lophioidei

The first lophioids were described by Linnaeus [3] (p. 236) and were classified among his *Amphibia Nantes* between two non-teleostean vertebrate genera: *Acipenser* and *Chimaera*. This classification was distinguished by “Spiracula lateralia” and “Pinnae natatoriae” meaning “lateral spiracles” and “swimming fins,” as the restricted gill openings of anglerfishes and allies were misinterpreted as the spiracles of cartilaginous fishes. Rafinesque [4] (p. 42) would recognize a Lofidi (Lophiidae) consisting of *Lophius piscatorius* that he allied with the Balastini (that consisted of *Balistes annularis* and *Capriscus porcus*, both synonyms of *Balistes capriscus*). The Lofidi and the Balastini, along with gobiesocids, congrids, chlopsids, and nettastomatids, would be grouped together within Rafinesque’s [4] (p. 41) “Seconda Divisone. Omnanchidi” that were described by “Branchie proviste di membrana branchiala, ma senza opercolo” [4] (p. 41) or “gills provided with gill membrane but without operculum. ” Cuvier [5] later published an influential study that grouped his examined representatives of the modern Antennariidae, Lophiidae, and Ogcocephalidae together with the Batrachoididae (toadfishes). He named this grouping within his “Acanthoptérygiens” as the “pectorales pédiculées” [5] (p. 249) or “spiny-rayed fishes whose long carpal bones [=radials] form a kind of arm that carries the pectoral [rays]. ” Günther [6] would place his examined representatives of the modern Antennariidae, Ceratiidae, Lophiidae, and Ogcocephalidae in a family named Pediculati that he allied with his Batrachidae (=Batrachoididae). This was supported by Regan [7] who classified the toadfishes as the suborder Batrachoidea and the anglerfishes and allies as the suborder Lophioidea in his Pediculati. Regan [7] grouped this Pediculati based on a series of features, emphasizing the presence of elongate pectoral radials, similarities in cranial anatomy, and the shared fusion of the hypurals into expanded plates. Later Regan [8], restricted the Pediculati to the anglerfishes and allies noting, “although the resemblances in the pectoral arch may be due to relationship, the differences in other characters are sufficient to keep them [Batrachoididae and Lophiodei] apart.” This revised classification was generally accepted throughout the 20th century, with some authors indicating a relationship within the same superorder [9–11] and even some early diagrams of relationships, predating modern phylogenetic systematics, suggesting a close relationship between the modern Batrachoididae and our Lophioidei (e.g., [12,13]).

During the middle of the 20^th^ century, the classification of fishes had become more similar to our modern groupings, notably in the influential work of Berg [14] who presented our Lophioidei adjacent to the Batrachoididae, indicating a close relationship. Greenwood et al. [9] circumscribed a Paracanthopterygii that included our Lophioidei and the modern Batrachoididae, Gadiformes (cods and allies), Gobiesocidae (clingfishes), and potentially the Percopsiformes (troutperches and allies). Their paracanthopterygians were grouped together based on 27 trends and features, ranging from modifications to the feeding mechanisms optimized for carnivory to the ceratohyal and epihyal being ankylosed. Rosen and Patterson [10] provided additional evidence for lophioid relationships and presented 32 characters that united the modern representatives of the Antennariidae, Chaunacidae, and deep-sea anglerfishes (Ceratioidei) to the Batrachoididae and the Gobiesocidae within “the Batrachoidiform Lineage” and, while noting differences in feeding, locomotion, jaw musculature, and the caudal skeleton among these taxa, further noted the lack of fossil evidence to support this clade.

Later Patterson and Rosen [15], proposed two synapomorphies for their Pediculati (Batrachoididae and our Lophioidei): (1) “the ventral gill arches converge on a very short copula, which is ossified very feebly or not at all” and (2) “the prezygapophyses of the first vertebra insert into hollow exoccipital bony tubes that are secondarily elongated, extending to or beyond the basioccipital condyle” [15].

Phylogenetic argumentation (albeit inexplicit) has played a crucial role in establishing support for the relationship between our Lophioidei and the Batrachoididae [16–20] based on morphological and behavioral data. In contrast, phylogenetic analyses of molecular data have consistently suggested alternative sister groups for the anglerfishes and their allies (early examples include [21–27]. In the first test with broad sampling, Miya et al. [22] hypothesized that the Lophioidei was sister to a clade composed of the triggerfishes and filefishes (Tetraodontoidei herein; Tetraodontiformes of Nelson et al. [1] and *Antigonia* (nested within their Percomorpha). Later Miya et al. [28], added the Batrachoididae to their analysis and recovered the toadfishes widely separated from the Lophioidei (i. e., the Batrachoididae was found sister to the Synbranchiformes near the base of the Percomorpha).

**Fig 2:**
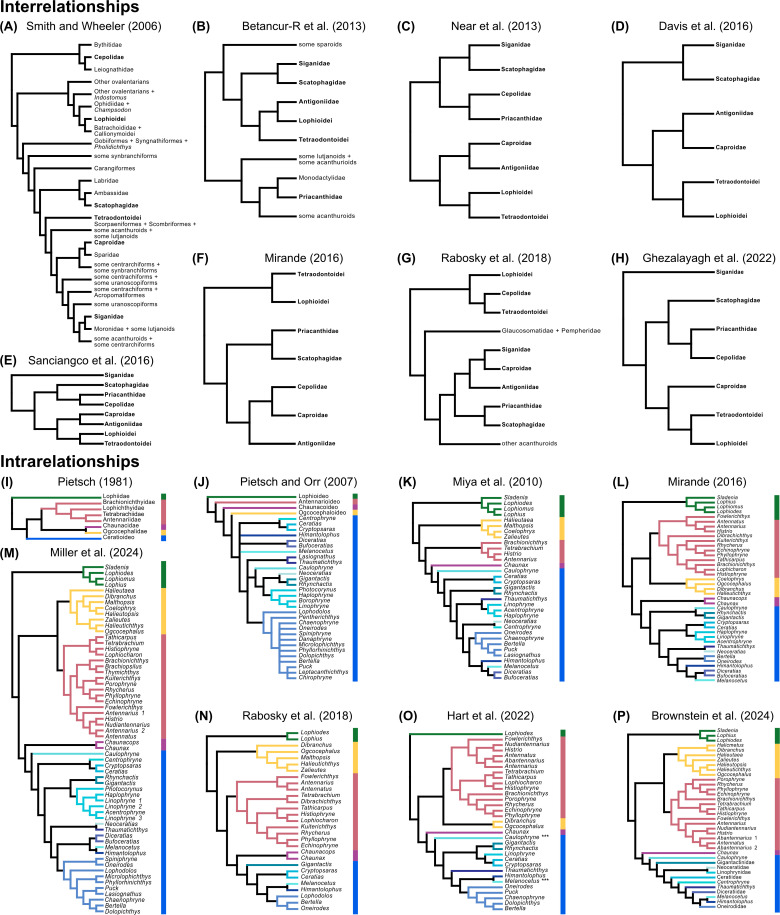
Prior phylogenetic hypotheses of inter and intraordinal relationships of anglerfishes (Lophioidei). Phylogenetic hypotheses and data type highlighting intraordinal relationships from studies that include two or more lophioid taxa, at least one tetraodontoid taxon, and four of the six other common lophioid allies (Antigoniidae, Caproidae, Cepolidae, Priacanthidae, Scatophagidae, and Siganidae) in their analyses: (A) mitochondrial and nuclear DNA sequence data, (B) mitochondrial and nuclear DNA sequence data, (C) nuclear DNA sequence data, (D) mitochondrial and nuclear DNA sequence data, (E) mitochondrial and nuclear DNA sequence data, (F) mitochondrial and nuclear DNA sequence data with morphology, (G) mitochondrial and nuclear DNA sequence and (H) ultraconserved element DNA sequence data. Phylogenetic hypotheses and data type highlighting infraordinal relationships from studies that include 20 or more genera in their analysis: (I) morphology, (J) morphology, (K) mitochondrial genomes, (L) mitochondrial and nuclear DNA sequence data with morphology, (M) exonic, mitochondrial, and nuclear DNA sequence data, (N) mitochondrial and nuclear DNA sequence data, (O) ultraconserved element DNA sequence data and (P) ultraconserved element DNA sequence data. Asterisks (***) in panel O indicate swapped taxa (see discussion: Ceratioideo).

Subsequent phylogenetic studies investigating the interrelationships of the Lophioidei would yield varying sister-group relationships for the Lophioidei (Fig 2). Six families, which include the Antigoniidae, Caproidae, Cepolidae, Priacanthidae, Scatophagidae, and Siganidae, and the Tetraodontoidei are the most common groups recovered near the Lophioidei [29–36], but these results have been extremely variable, even when most of the taxa are present in the analysis (Fig 2). The relationships of these acanthuriform taxa are barely discussed in detail, and only a handful of these studies have included all six families and tetraodontid taxa [31,35,36] to fully address their relationships (Fig 2).

### Intrarelationships, habitat, and depth variation within the Lophioidei

The monkfishes and goosefishes (Lophioideo; Fig 1A) are found in benthic/demersal environments from shallow to mid depths on the outer and upper continental slopes. These fishes typically reside on muddy, sandy, or rocky substrates that conceal their flattened bodies. A single-family, Lophiidae, and four genera comprise the clade [2]. The monkfishes have been historically recognized as a separate clade and are typically presented as the stem group to the rest of the anglerfishes and allies [16,17,19,34,36–40], with an exception by Shedlock et al. [41] who recovered the clade as the sister group to the coffinfishes and deep-sea anglerfishes (Fig 2).

The frogfishes (Antennarioideo; Fig 1B and 1C) are found primarily in benthic/demersal shallow depths of the ocean, except for *Histrio histrio*, which is a pelagic frogfish found living on floating sargassum seaweed, and *Antennarius biocellatus*, which ventures into freshwater and brackish environments [18]. These fishes have a laterally compressed corpulent build for traversing and blending into muddy, sandy, or rocky benthic/demersal substrates. There are seven families in the Antennarioideo: Antennariidae, Brachionichthyidae, Histiophrynidae, Lophichthyidae, Rhycheridae, Tathicarpidae, and Tetrabrachiidae comprising 23 genera [38]. The placement of the frogfish clade among the anglerfishes and allies has varied (Fig 2): sister to Chaunacidae + Ogcocephalidae [16]; sister to all non-lophiid members of the Lophioideo [17,19]; sister to Chaunaxidae + Ceratioideo [36,37]; sister to Ogcocephalidae [34,38–40] across multiple studies with the exception of Miller et al. [40] who recovered a paraphyletic Antennarioideo.

The batfishes (Ogcocephaloideo; Fig 1D and 1E) are found primarily in shallow to deep-water benthic/demersal habitats [42] and are dorsoventrally flattened, with some species possessing an elongated upturned snout. The distinctive profiles of batfishes exhibit a captivating range of shapes, characterized by remarkable variability and an intriguing spectrum of morphologies, including triangular, ovular/circular, and even curved forms viewed from above. A single-family, Ogcocephalidae, and ten genera comprise the clade [2]. The family has been consistently recovered as monophyletic [19,36–41], but the placement of the family among the anglerfishes and allies has been highly variable and ranges from sister to each of the individual non-lophiid infraorders to sister to all non-lophiid anglerfishes (Fig 2).

The coffinfishes (Chaunacoideo; Fig 1F) are found in deep-water, benthic/demersal habitats on the outer continental shelf and the upper continental slope. The bodies of these fishes are globular and slightly elongated. The clade is comprised of a single family, Chaunacidae, which comprises ten genera [2]. The family has been consistently recovered as monophyletic [34,36–40], but the placement of the family among the anglerfishes and allies has varied in placement (Fig 2) with results a sister-group relationship with the batfishes [16], batfishes and deep-sea anglerfishes [17], deep-sea anglerfishes [36–41], or the frogfishes [34].

The deep-sea anglerfishes (Ceratioideo; Fig 1G–1J) are found in deep-water habitats from the mesopelagic to the abyssopelagic zone. Except for *Thaumatichthys*, these fishes occupy pelagic habitats. In addition, most deep-sea anglerfishes undergo a variety of vertical migrations associated with larval and metamorphosed development [43,44]. These fishes are highly variable in body shape, ranging from elongated to globular. The infraorder is comprised of 11 families: Caulophrynidae, Centrophrynidae, Ceratiidae, Diceratiidae, Gigantactinidae, Himantolophidae, Linophrynidae, Melanocetidae, Neoceratiidae, Oneirodidae, and Thaumatichthyidae. This infraorder comprises 35 genera and is the most species-rich infraorder within the Lophioidei, containing 311 valid species [2]. The placement of the deep-sea anglerfish clade within the Lophioidei has been variable [16,17,19,34], with several authors [36–41] recovering the Ceratioideo sister to the Chauncoideo (Fig 2). Further, the relationships among the deep-sea anglerfish families have had little consistency (see Fig 2: [16,19,34,36–40].

### Body-shape variation

The Lophioidei is an ideal lineage for studying the evolution of body shape in marine environments, as they have a variety of body-shape morphologies that vary across a group that is found in inshore shallow waters and the deep sea in both benthic/demersal and pelagic habitats. The transition from one environment to a new environment typically coincides with a change in body shape as a group adapts to its new habitat [40,45–47]. Batfishes and goosefishes, for example, occupy similar benthic/demersal habitats and have dorsally flattened bodies; in contrast, frogfishes typically have stocky, round bodies. Certain anglerfishes in deep-water habitats display globular body morphologies such as the coffinfishes (*Chaunacops*, *Chaunax*) and deep-sea anglerfishes (e.g., *Caulophryne*, *Himantolophus*, *Melanocetus*, *Oneirodes*). These contrast with the elongate bodies typical of most deep-sea fishes [47–52], and such variation contradicts the notion that there is a universal selective pressure on all deep-sea fishes to reduce their silhouette, whether through body elongation [53], vertical orientation, or bioluminescent counter-shading [54,55]. Previous investigations into the evolution of body shape in marine environments have used geometric morphometric methods to quantify body-shape variation in response to different and varying habitats [40,45,46,48,50,52,55–61].

To date, no phylogenetic analyses have incorporated morphological, mitochondrial, and genome-scale nuclear (e.g., ultraconserved elements [UCEs]) data to produce a total-evidence phylogeny for the Lophioidei to infer the evolutionary relationships of this lineage of fishes. In this study, we explore several lines of inquiry to better understand the evolutionary relationships and morphological adaptations of the Lophioidei and their related groups. First, we use a total-evidence approach, which in our study refers to the combination of genome-scale molecular data (UCEs and Sanger DNA fragments/mitogenomes) and explicit morphological data, to infer the evolutionary relationships of this clade (see [62–64]). This approach aims to address the following questions: (1) What are the inter- and intrarelationships of the Lophioidei? Further, we aim to evaluate the status of the synapomorphies for the Lophioidei, its sister group, and its major groups: (2) What are the synapomorphies of the Lophioidei, the lophioid infraorders, and the acanthuriform groups allied with the lophioids? Next, we investigate the depth and habitat transitions of the anglerfishes and allies using ancestral character-state reconstructions: (3) What are the habitat and depth preferences of the Lophioidei and its major groups? Finally, we explore the variability, breadth, and changes in lateral body-shape morphology in the lophioid suborders and across the transitions across different habitats and depths: (4) Are there quantifiable differences in body shape found within the Lophioidei?

## Materials and methods

For this study, the anglerfishes and allies (Lophioidei) are treated as a suborder following the ordinal classification of Davis et al. [33] and Smith et al. [65]; in this classification, this clade is within an expanded Acanthuriformes. Infraordinal and subordinal endings follow Girard et al. [63] and Tyler et al. [66] and all family, genus, and species names follow Fricke et al. [2] and van Der Laan and Fricke [67] unless modified in this study. We are following the clade-level nomenclature adopted herein because it aligns with recent phylogenetic evidence and reflects a modern understanding of evolutionary relationships within the group, providing a standardized Linnean framework for discussing the taxonomy and systematics of anglerfishes and their allies.

### Taxon sampling

Museum acronyms follow Sabaj [68]. Physical examinations of specimens used in this study for character state coding and geometric morphometric analyses (S1 Table) included material from the Field Museum of Natural History (FMNH), Natural History Museum of Los Angeles County (LACM), and University of Kansas Biodiversity Institute and Natural History Museum (KU). Additional photographs of anglerfish specimens were provided by request from the Australian Museum (AMS), Museum für Naturkunde (ZMB), and Muséum national d’Histoire naturelle (MNHN). Additional photographs of adult anglerfish species were included from prior peer-reviewed publications and websites (S2 Table). Fish tissue samples came from the following institutions: American Museum of Natural History (AMNH), Biodiversity Research Museum, Chinese Academy of Sciences (ASIZ), Commonwealth Scientific and Industrial Research Organization (CSIRO), FMNH, KU, Harvard University Museum of Comparative Zoology (MCZ), MNHN, and Scripps Institution of Oceanography, Marine Vertebrate Collection (SIO), as well as the collection of Agnès Dettaï at MNHN (S3 Table).

Taxonomic sampling included all families and most genera within the Lophioidei covering the five infraorders: Antennarioideo, Ceratioideo, Chaunacoideo, Lophioideo, and Ogcocephaloideo. For the UCE-only dataset, a total of 35 terminals were analyzed with 21 anglerfish species representing 14 genera, 11 families, all infraorders, and 13 outgroup taxa. We included *Antigonia capros*, *Antennarius striatus* from Alfaro et al. [69] and *Ogcocephalus cubifrons* from Hart et al. [38] which was originally identified in their study as *Ogcocephalus radiatus*. Outgroups for the UCE analysis included 13 acanthuriform genera. *Morone saxatilis* was chosen as the root because it has been consistently recovered as a stem representative of the Acanthuriformes [31,33,65]. To increase taxon sampling, a UCE+mitochondrial DNA dataset was assembled that included, when available, mitochondrial genomes, newly extracted partial mitochondrial genomes from the UCE data, Cytochrome c oxidase subunit I, and/or 16S from multiple studies [22,25,37,38,42,70–82] and several unpublished sources (S3 Table).

To further increase the taxon and character sampling, 100 morphological characters were used to generate a total-evidence matrix (S4 Table). Characters 1–88 are from Pietsch and Orr [19] and allowed for the addition of the genera *Borophryne*, *Chirophryne*, *Danaphryne*, *Pentherichthys*, *Phyllorhinichthys*, *Photocorynus*, and *Spiniphryne* from the morphological matrix from Pietsch and Orr [19]. Characters 89–100 were sourced from the following: character 89 from Tyler and Sorbini [83]; character 90 based on the description of the character by Rafinesque [4]; character 91 from Tyler [66]; characters 92 and 93 from Pietsch [16] and Tyler and Sorbini [83]; character 94 from Regan [7] and Tyler and Sorbini [83]; character 95 from Regan [7]; character 96 modified based on the description of the character by Fink’s [84]; character 97 from Pietsch [16] and Rasquin [85]; character 98 from Regan [7]; character 99 based on the description of the character by Cuvier; and character 100 is proposed in this study. Characters 1–88 were coded as ambiguous for non lophioid outgroup taxa and characters 89–100 were coded based on examination of clear and stained specimens (S1 Table and descriptions in the previous literature [4,16,17,43,66,72,77,84,86–113] (see discussion for specific citations relevant per character). This molecular and morphological dataset includes all five lophioid infraorders, all 21 families, and 67 of 74 lophioid genera.

### DNA extraction

Fish tissues were preserved in 70–95% ethanol or stored cryogenically prior to the extraction of DNA. DNA extractions were taken from muscular tissue samples or fin clips of 34 fishes (21 lophioid terminals and 13 outgroup species) using either a DNeasy Tissue Extraction Kit (Qiagen) or the Maxwell® RSC Whole Blood DNA Kit (Promega) following the manufacturer’s extraction protocols (with the replacement of the blood DNA kit’s lysis buffer with Promega’s tissue lysis buffer). When necessary, multiple samples were combined using a Thermo Fisher SpeedVac Concentrator to a 102 µl volume. Two microliters of the raw or concentrated extracts were quantified using a Qubit fluorometer using the dsDNA BR Assay kit following the manufacturer’s protocol. Following quantification, the samples were sent to Arbor Biosciences (Ann Arbor, MI) at a 100 µl volume for library preparation (e. g., DNA shearing, size selection, cleanup), target capture (using the 500 UCE actinopterygian loci probe set [114]; enrichment, sequencing using an Illumina HiSeq 2500 or NovaSeq 6000, and demultiplexing of samples.

### DNA amplification, sequencing, and assembly

UCE data were received in a FASTQ file format. The FASTQ data that included sequences from multiple runs was cleaned of indices and adapters using illumiprocessor [115] and Trimmomatic [116]. Once cleaned, the reads were assembled into contigs using SPADES v3. 15. 4 [117]. UCE loci were identified for each species using the actinopterygian probe set and assembled into a relational database using PHYLUCE v1. 7. 2 [118] and LASTZ v1. 02. 00 [119] set at 80% minimum coverage and 80% minimum identity for finding UCEs. PHYLUCE v1. 7. 2 [118] was used to create a database of UCE loci by taxon and then to construct FASTA files of the UCE data. Cleaned UCE data were submitted to GenBank (S1 Table for accession numbers). The extracted UCE data were aligned with MAFFT [120] with a data matrix that included only contigs found in at least 65% of the included taxa. For the 35 terminals that were sampled for UCEs, a total of 462 aligned UCE fragments were concatenated for a total length of 302,543 bps. Following the extraction of the UCEs, we also used MitoFinder [121] to extract available mitogenomic data from the cleaned DNA sequence read archives (SRA) for the species that did not have previously published mitogenomic data. Mitochondrial data (S3 Table) were aligned using MAFFT [120] to produce a combined dataset of UCE fragments and mitochondrial data that included 92 terminals and 333,766 bps. SRA data were mapped to previously published lophioid Cytochrome c oxidase subunit I or mitochondrial genomes using the ‘map to reference’ function in Geneious 2024. 0. 7 [122]. The Geneious tool was used for the ‘mapper’ function, the ‘medium sensitivity/ fast’ setting was used for the ‘sensitivity’ function, and the ‘iterate up to 10 times’ setting was used for the ‘fine tuning’ function. Mapped SRA data were then blasted on GenBank [123] and Bold Systems [124] for taxon identification.

### Phylogenetic analyses

The UCE data included 35 terminals and the UCE fragments were partitioned using SWSC-EN [125]. Each species-specific UCE locus and surrounding DNA was split into regions of left flanking, right flanking, and core by rate of evolution. These UCE regions were then submitted to PartitionFinder2. 1. 1 [126–128] to identify the best-fitting nucleotide substitution model for each data partition and to combine partitions as appropriate. We similarly used PartitionFinder2. 1. 1 [126–128] for the combined UCE and mitogenomic dataset that included a total of 92 terminals. PartitionFinder2. 1. 1 [126–128] identified 389 subsets for the UCE-only dataset and 390 for the combined dataset with the mitochondrial data (S5 Table) and identified the preferred substitution model for each partition. The UCE and UCE+mitochondrial datasets (S6 Table) were analyzed using maximum likelihood with IQ-TREE v2. 2. 2. 6 [129] applying the partition and substitution models (S5 Table) identified by PartitionFinder2. 1. 1. The molecular and morphological dataset was analyzed in IQ-TREE v2. 2. 2. 6 [129] used the same models for the molecular data and added an additional anatomical partition that used the MK+ASC model of character evolution. For each dataset, the analysis began with 20 independent replicates. Following the 20 replicates, the results were combined into a single starting tree and submitted to more thorough analysis using the commands -nbest 25, -allnni, -pers 0. 6, and -nstop 250. The tree with the highest likelihood values from these resulting analyses are presented in the results. Bootstrap replicates (1,000 ultrafast) were also calculated using IQ-TREE v2. 2. 2. 6 [129] for each dataset and are denoted on the nodes of the respective hypotheses.

### Character optimization

The tree topology of the total-evidence phylogeny was used to evaluate the proposed synapomorphies for the Lophioidei, its infraorders, and the included lophioid acanthuriform allies. We optimized the morphological matrix (S4 Table) using WinClada v1. 00. 08 [130] and Mesquite v3. 81 [131]. In WinClada, only unambiguous parsimony character-state optimizations were visualized; in Mesquite, optimizations were performed using parsimony for morphological character-state transformations.

To explore habitat and depth character evolution, we used the total-evidence phylogeny to infer depth and habitat transitions. Depth data for each terminal was based on museum collection data from FishNet2. The mean depth for each species was calculated (following the procedure of Smith et al. [132]) and assigned the following character states for depth ranges: (0) found in both fresh and epipelagic marine habitats, (1) predominantly found in the epipelagic zone (≤ 200 m), (2) predominantly found in the mesopelagic zone (200–1,000 m), (3) predominantly found in the bathypelagic zone (1,000–3,000 m), and (4) predominantly found in the abyssopelagic zone (3,000–6,000 m). Character states for benthic and/or demersal vs. pelagic habitat references were coded based on descriptions from the literature [19,43,75,89,102,104,133–140] and ROV videos that included: (0) predominantly found near the seafloor (benthic/demersal) and (1) predominantly found in the open water column (pelagic). Taxa were treated and coded the same if either benthic/demersal or demersal. See S7 Table for character matrix. Ancestral character-state reconstructions were based on a parsimony optimization using the total-evidence phylogeny in Mesquite [131].

### Geometric morphometrics and statistical analyses

Adult anglerfish species used in geometric morphometric analysis included material from AMS, FMNH, LACM, MNHN, and ZMB (S1 Table). Additional photographs of adult anglerfish species were included from prior peer reviewed publications and websites listed in S2 Table. Larval and juvenile forms as well as ceratioid males were excluded from the analysis due to their smaller size and distinct morphology. In total, 111 specimens representing 102 species, 59 of 74 genera, and all families were examined. Specimens physically examined were photographed under white lighting conditions using a Canon EOS Rebel T7i DSLR camera equipped with a Canon EF-S 60mm f/2. 8 Compact Macro USM lens while positioned on their lateral side facing left.

**Fig 3:**
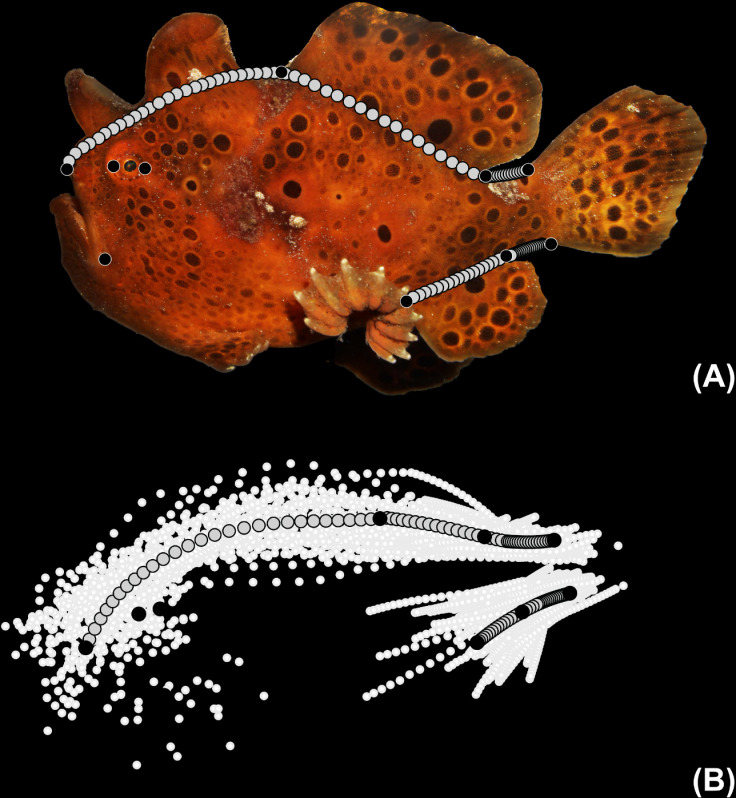
Geometric morphometric landmark positions. (A) Position of fixed homologous landmark (black) and sliding semi-landmark (gray) locations used in this study. (B) Consensus anglerfish and allies body shape from geometric morphometric analysis.

A landmark-based geometric morphometric approach was used to quantify lophioid body shapes. Using the R package stereomorph [141], 10 homologous landmarks (black circles in Fig 3A) were placed at the (1) anteriormost tip of the premaxilla, (2) posteriormost tip of the maxilla, (3) anterior margin of the eye, (4) posterior margin of the eye, (5) anterior insertion of the soft dorsal fin (this does not incluce the illicium or any cephalic spines), (6) posterior insertion of the soft dorsal fin, (7) dorsal insertion of the caudal peduncle, (8) ventral insertion of the caudal peduncle, (9) posterior insertion of the anal fin, and (10) the anterior insertion of the anal fin. One hundred sliding semi-landmarks were distributed across five defined curves along anglerfish body margins (Fig 3A) as used in prior studies [47,62,142]. To prevent the inclusion of shape variation due to stomach fullness [143] or stomachs altered due to dissections, we excluded semi-landmarks between the lower jaw and the anterior insertion of the anal fin (Fig 3A).

Body-shape analyses follow Maile et al. [47] and Martin et al. [52]. The R package geomorph v. 4. 0. 5 [144] was used to perform a generalized Procrustes analysis (GPA) using bending energy for semi-landmark alignment. Specimen-shape values and centroid sizes were averaged by genus (see S8 Table for genus-level labels). The total-evidence phylogenetic analysis of the Lophioidei was used to incorporate average Procrustes variables in a phylogenetic regression consisting of 10,000 iterations using the geomorph function ‘procD. pgls’ [144] to test the allometric effect between body shape and specimen size. Under residual randomization, regression resampling was calculated using residual shape values that were obtained from a reduced model and then randomly assigned to the original linear model. After removing the observed variation in shape caused by differences in specimen size and phylogenetic influence using residuals from this technique, a principal component analysis (PCA) was plotted to visualize the shape data across anglerfishes and allies. We calculated morphological disparity using the geomorph function ‘morphol. disparity’ [144] to compare potential differences in the amount of body-shape variability among anglerfishes, anglerfish infraorders, depth preferences, and pelagic or benthic/demersal preferences. Alpha for significance was set to 0.05.

## Results

**Fig 4:**
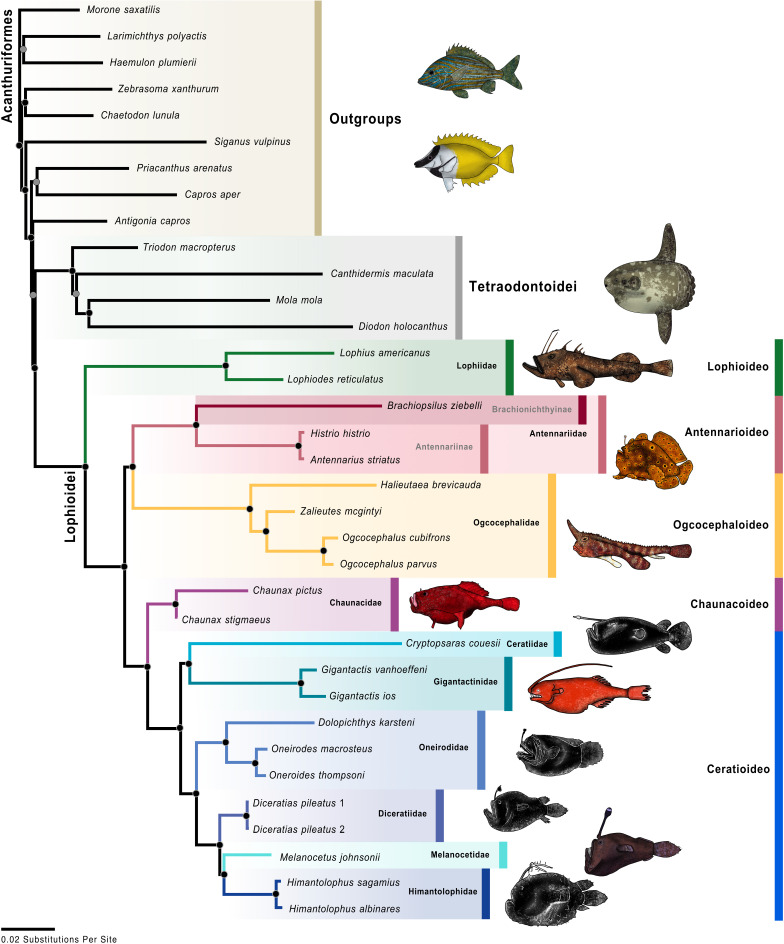
UCE maximum-likelihood tree. Colored circles on nodes indicate bootstrap values as follows: white 50% to 69%, gray 70% to 89%, black 90% to 100% based on 1,000 ultrafast bootstrap replicates. The color scheme for infraorders and families corresponds to Figs 1 and 2. Fish illustrations by Alex Maile.

**Fig 5:**
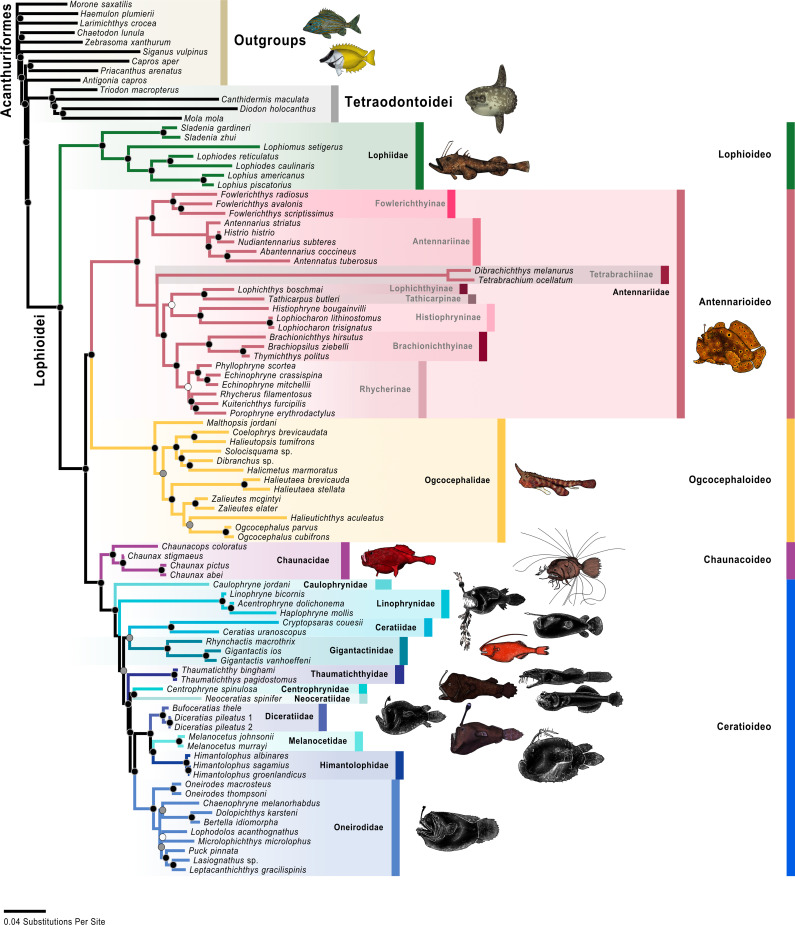
Molecular maximum-ikelihood tree. Colored circles on nodes indicate bootstrap values as follows: white 50% to 69%, gray 70% to 89%, black 90% to 100% based on 1,000 ultrafast bootstrap replicates. The color scheme for infraorders and families corresponds to Figs 1 and 2. Fish illustrations by Alex Maile.

**Fig 6:**
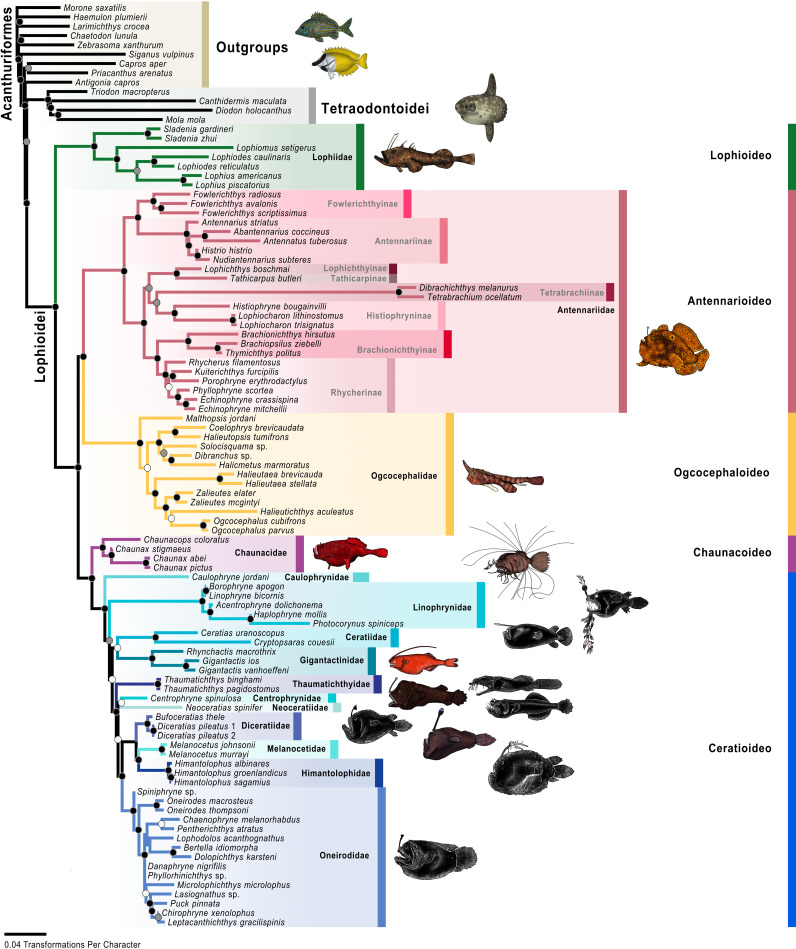
Total-evidence maximum-likelihood tree. Colored circles on nodes indicate bootstrap values as follows: white 50% to 69%, gray 70% to 89%, black 90% to 100% based on 1,000 ultrafast bootstrap replicates. The color scheme for infraorders and families corresponds to Figs 1 and 2. Fish illustrations by Alex Maile.

The analysis of the UCE data resulted in a single optimal tree (Fig 4) with a likelihood score of -2022703. 073. Twenty-eight nodes (of the possible 32 nodes; 86. 5%) were strongly supported or better (>90% bootstrap values), 32 nodes (100%) were well supported or better (>70% bootstrap values), and 32 nodes (100%) were moderately supported or better (>50% bootstrap values). The analysis of the UCE data combined with mitochondrial data, or “molecular analysis,” resulted in a single optimal tree (Fig 5) with a likelihood score of -2472345. 236. Seventy-seven nodes (of the possible 89 nodes; 86. 5%) were strongly supported or better, 84 nodes (94. 4%) were well supported or better, and 86 nodes (96. 6%) were moderately supported or better. The analysis of the molecular and morphological matrix or “total-evidence analysis,” resulted in a single optimal tree (Fig 6) with a likelihood score of -2473999. 509. Seventy-five nodes (of the possible 96 nodes; 78. 1%) were strongly supported or better, 84 nodes (87. 5%) were well supported or better, and 92 nodes (95. 8%) were moderately supported or better.

### Intrarelationships of the Lophioidei

**Fig 7:**
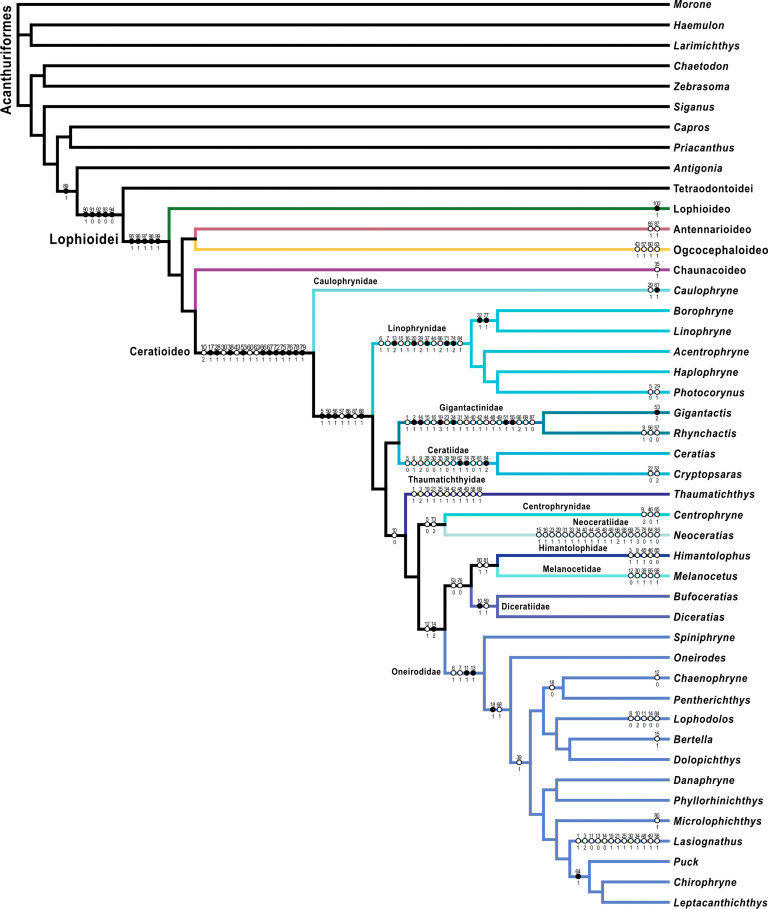
Morphological character optimization on the total-evidence phylogeny of the Lophioidei, Tetraodontoidei, and outgroups. Total-evidence phylogenetic analysis topology of Lophioidei and outgroups. Morphological characters optimized onto each node are represented by a circle with the corresponding character number listed above and the corresponding character state listed below. Circles with black fill-in are unique and unreversed characters. Circles with white fill-in are inferred to have transformed multiple times on the phylogeny.

The results of all three analyses (UCE, molecular, and total evidence) recovered the same evolutionary relationships among the infraorders (Figs 4–6), and their bootstraps were all strongly supported. The Lophioideo (Lophiidae) was the sister infraorder to a clade composed of the Antennarioideo (Antennariidae), Ceratioideo, Chaunacoideo (Chaunacidae), and Ogcocephaloideo (Ogcocephalidae). The Antennarioideo and Ogcocephaloideo were sister groups, and together these were sister to a clade consisting of the Ceratioideo and Chaunacoideo, which were also sister groups. In the total-evidence analysis, the Lophioidei was supported by five morphological synapomorphies (characters 95–99; Fig 7), the Lophioideo was supported by a single morphological synapomorphy (character 100; Fig 7), the Antennarioideo was supported by two morphological synapomorphies (characters 85 and 87; Fig 7), the Ogcocephaloideo was supported by four morphological synapomorphies (characters 43, 57, 60, and 63; Fig 7), the Chaunacoideo was supported by a single morphological synapomorphy (character 35; Fig 7), and the Ceratioideo was supported by 16 morphological synapomorphies (characters 10, 17, 28, 30, 38, 43, 53, 60, 63, 66, 67, 72, 75, 76, 78, and 79; Fig 7).

In the molecular and total-evidence analyses (Figs 5 and 6), we recovered a monophyletic Antennarioideo consisting of the Antennariidae, Brachionichthyidae, Histiophrynidae, Lophichthyidae, Rhycheridae, Tathicarpidae, and Tetrabrachiidae. Within the Antennariidae, there were two clades, one consisting of *Fowlerichthys* and a second that includes all other antennariids. Given our consistent separation of *Fowlerichthys* on a notably elongate branch from all other antennariids, the similarity of results to recent analyses [34,36,38–40,145], and the occasional recovery of *Fowlerichthys* sister to all non-antennarid antennarioids, we recommend changes to the classification of the frogfishes and allies (Antennarioideo) that ensures taxonomic stability. There are two classifications that are defensible. The first option entails describing another frogfish family for species in *Fowlerichthys* that would join Histiophrynidae (7 genera; [71], Rhycheridae (1 genus; [38], and Tathicarpidae (1 genus; [38] as new families described out of the Antennariidae since 2010. This option divides this consistently recovered clade into seven or eight smaller families, each comprising one to seven genera. The second option involves the recognition of a larger Antennariidae that is equivalent in composition to the Antennarioideo, encompassing all antennariids (including *Fowlerichthys*), brachionichthyids, histiophrynids, lophichthyids, tathicarpids, tetrabrachiids, and rhycherids. The recognition of multiple families would highlight the richness and diversity within the clade, while the recognition of a single larger family would ensure accurate family-level identification by conservationists, divers, and ichthyologists as each new frogfish family requires additional expertise for accurate identification.

**Fig 8:**
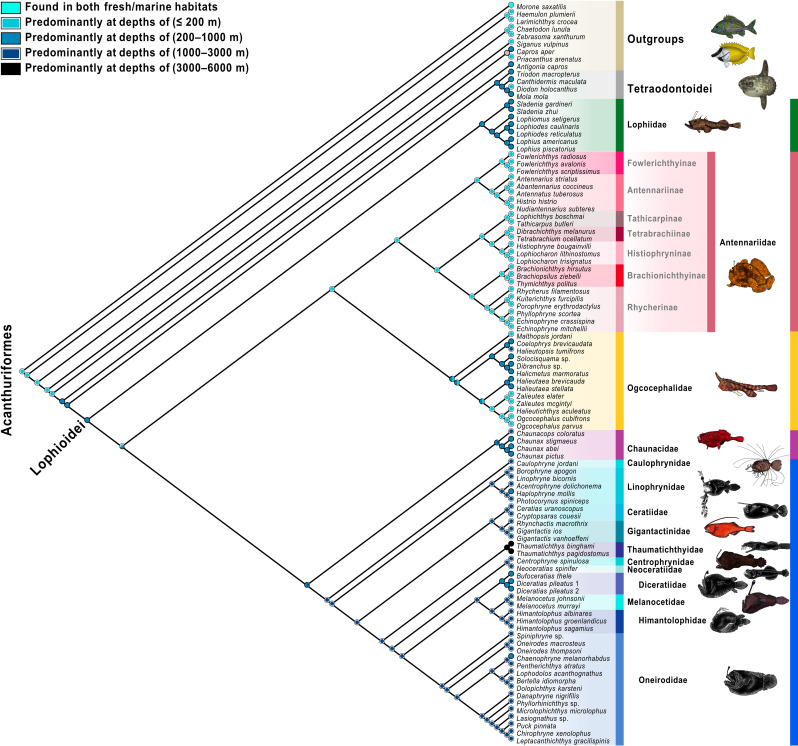
Anglerfishes and allies habitat transitions. Parsimony character-state analysis of habitat transitions of anglerfishes based on the total-evidence maximum-likelihood phylogeny. The color scheme for infraorders and families corresponds to Figs 1 and 2. Fish illustrations by Alex Maile.

**Fig 9:**
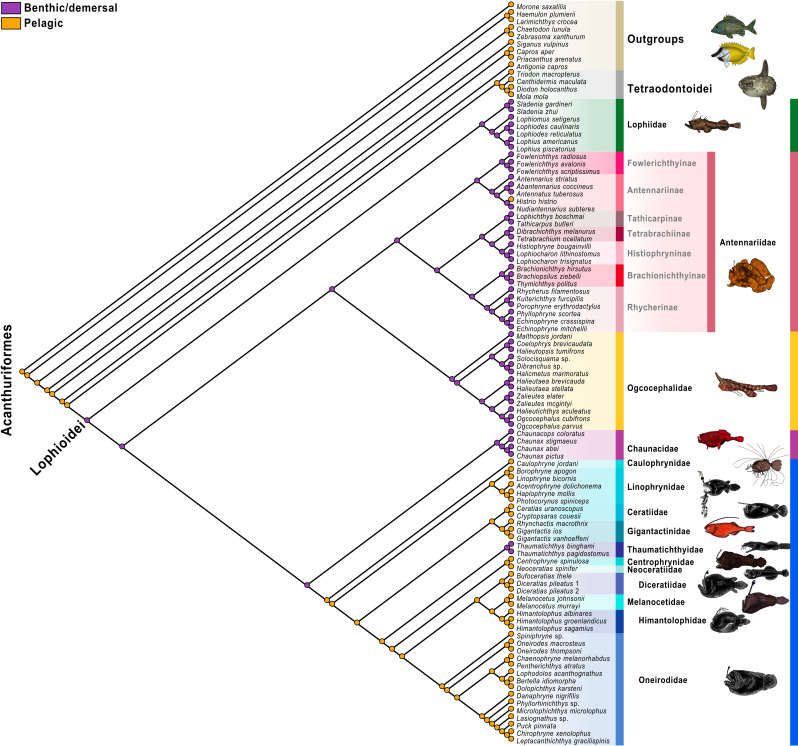
Anglerfishes and allies depth transitions. Parsimony character-state analysis of benthic/demersal or pelagic preferences of anglerfishes based on total-evidence maximum-likelihood phylogeny. The color scheme for infraorders and families corresponds to Figs 1 and 2. Fish illustrations by Alex Maile.

We agree with Near and Thacker [146] and recommend placing the Brachionichthyidae, Histiophrynidae, Lophichthyidae, Rhycheridae, Tathicarpidae, and Tetrabrachiidae in the synonymy of the Antennariidae. Further, we recommend placing the Brachionichthyidae, Histiophrynidae, Lophichthyidae, Rhycheridae, Tathicarpidae, and Tetrabrachiidae in a subfamilial classification with antennariids distributed across the Antennariinae, Brachionichthyinae, Lophichthyinae, Histiophryninae, Rhycherinae, Tathicarpinae, Tetrabrachiinae, and a new subfamily for *Fowlerichthys*. Given that the results presented here are based on this revised taxonomy, which treats several previously named families as subfamilies, this classification will be used for the remainder of the paper unless otherwise noted. The revised Antennariidae and its subfamilies will be used for the remainder of this paper unless otherwise noted and is used in the figures (Figs 4–6 and 8 and 9).

**Fowlerichthyinae** new subfamily Maile, Smith, and Davis
urn:lsid:zoobank.org:act:D072BAFC-E131-4DDD-B976-7FF0E59C2D18
**Type genus.** *Fowlerichthys* Barbour, 1941.
**Species included.** *Fowlerichthys avalonis* (Jordan and Starks, 1907), *F. ocellatus* (Bloch and Schneider, 1801), *F. radiosus* (Garman, 1896), *F. scriptissimus* (Jordan, 1902), and *F. senegalensis* (Cadenat, 1959).
**Diagnosis.** As described by Pietsch and Arnold [102] (p. 664), Fowlerichthyinae can be diagnosed by “… a character unique among lophiiforms: all five soft rays of the pelvic fins are bifurcate, in contrast to all other antennariids, which have, at most, only a single bifurcate pelvic-fin ray.”

In the molecular and total–evidence analyses (Figs 5 and 6), the Fowlerichthyinae was sister to the Antennariinae. Consequently, the scope of the Antennariinae is now limited to encompass species within the genera *Abantennarius*, *Antennarius*, *Antennatus*, *Histrio*, and *Nudiantennarius*. Together Antennariinae and Fowlerichthyinae are the sister group to a clade consisting of the Brachionichthyinae, Histiophryninae, Lophichthyinae, Rhycherinae, Tathicarpinae, and Tetrabrachiinae. Between the molecular (Fig 5) and total–evidence analyses (Fig 6), the Tetrabrachiinae varies in its position in the clade consisting of the Histiophryninae, Lophichthyinae, Tathicarpinae, and Tetrabrachiinae. In the molecular analysis, Tetrabrachiinae was sister to the Brachionichthyinae, Histiophryninae, Lophichthyinae, Rhycherinae, and Tathicarpinae, while in the total-evidence analysis (Fig 6), it was found sister to the Histiophryninae, which together were the sister group to a clade of the Lophichthyinae and Tathicarpinae. In the molecular and total-evidence analyses, Lophichthyinae was sister to the Tathicarpinae, and in the molecular analysis, these two subfamilies were resolved sister to the Histiophryninae. The Brachionichthyinae was sister to the Rhycherinae in the molecular and total-evidence analyses. Together, this clade was sister to the clade containing the Histiophryninae, Lophichthyinae, and Tathicarpinae, except for the total-evidence analysis where Tetrabrachiinae was also included in this clade.

In the molecular and total-evidence analyses (Figs 5 and 6), the Oneirodidae was sister to a clade consisting of the Diceratiidae, Himantolophidae, and Melanocetidae. Within this clade, the Diceratiidae was sister to the Himantolophidae and Melanocetidae. Together these families were the sister group to a clade composed of the Centrophrynidae and Neoceratiidae. These five families were sister to the Thaumatichthyidae. These six families were recovered as the sister group to a clade consisting of the Ceratiidae and Gigantactinidae. Finally, Linophrynidae was sister to those eight families, and Caulophrynidae was recovered at the base of the Ceratioideo.

### Depth and habitat reconstructions

We conducted an ancestral-state reconstruction of the anglerfishes and their allies (Figs 8 and 9) to examine the evolutionary history and depth transitions of its major clades. The tetraodontoid sister group was inferred to occupy depths between 200–1,000 m. The common ancestors of the Lophioidei and the Lophioideo were inferred to be found at depths ranging from 200 to 1,000 m. A single transition to depths <200 m was recovered for the Antennarioideo and several ogcocephaloid genera (*Halieutichthys*, *Malthopsis*, *Ogcocephalus*, and *Zalieutes*; Fig 8). We identified that two transitions to depths of 200–1,000 m from depths of <200 m occurred in the ancestors of the ogcocephalid genera *Coelophrys*, *Dibranchus*, *Halicmetus*, *Halieutopsis*, and *Solocisquama* and independently in *Halieutaea* (Fig 8). A single transition to depths of 1,000–3,000 m from depths of 200–1,000 m occurred in the ancestor of the clade composed of the Chaunacoideo and Ceratioideo. Four independent transitions to depths of 200–1,000 m from depths of 1,000–3,000 m are found in (1) the chaunacoid genus *Chaunax*, (2) the linophrynid genus *Haplophryne*, (3) the family Diceratiidae, and (4) the oneirodid genus *Chaenophryne* (Fig 8). A single transition to depths of 3,000–6,000 m from depths of 1,000–3,000 m occurred in *Thaumatichthys* (Fig 8).

We conducted an ancestral-state reconstruction of the anglerfishes and their allies (Fig 9) to infer the evolutionary history of benthic/demersal vs. pelagic habitats within the Lophioidei. The sister group to the Lophioidei, the Tetraodontoidei, was inferred to ancestrally occupy a pelagic habitat [133,136,145,147]. The common ancestor of the Lophioidei was inferred to be found in benthic/demersal spaces (Fig 9). A single transition to benthic/demersal spaces from pelagic spaces was found in the Thaumatichthyidae (Fig 9). Two independent transitions to pelagic spaces from benthic/demersal spaces were found in (1) the antennariid genus *Histrio* and (2) the ancestor of the Ceratioideo (Fig 9).

### Variation and body-shape disparity across the anglerfishes and allies

**Fig 10:**
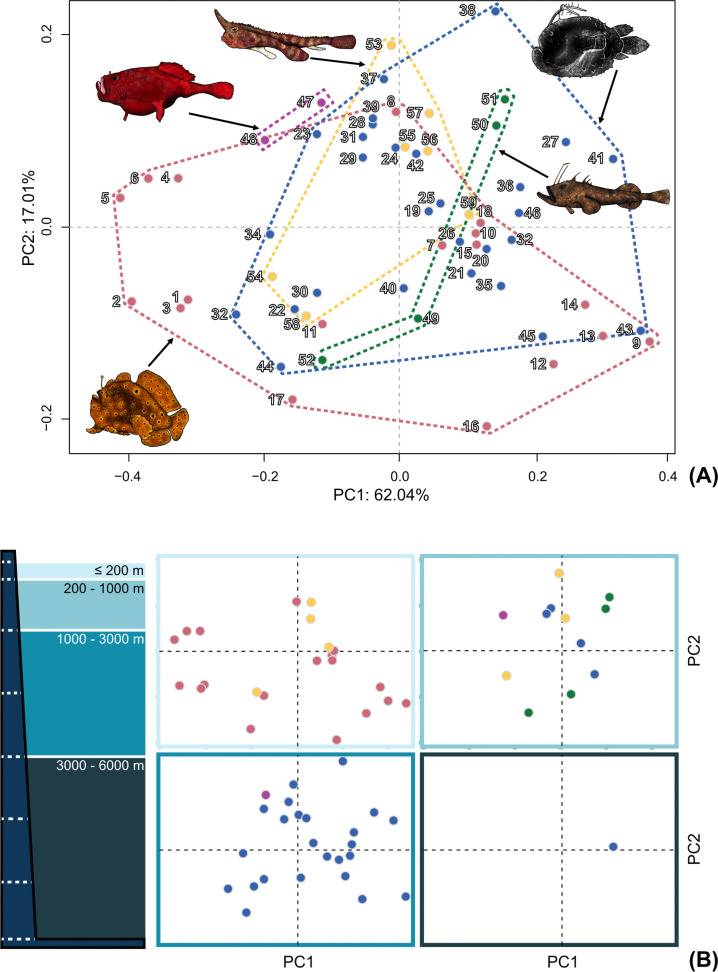
Relative warp analysis of anglerfish body shape depicting PC1 + PC2. (A) Relative warp analysis consisting of 11 homologous landmarks and 19 sliding landmarks. Circle positions represent the average location in the morphospace for each genus. See S8 Table for genus-level labels. (B) Relative warp analysis organized by depth range. The color scheme for infraorders and families corresponds to Figs 1 and 2. Fish illustrations by Alex Maile.

**Fig 11:**
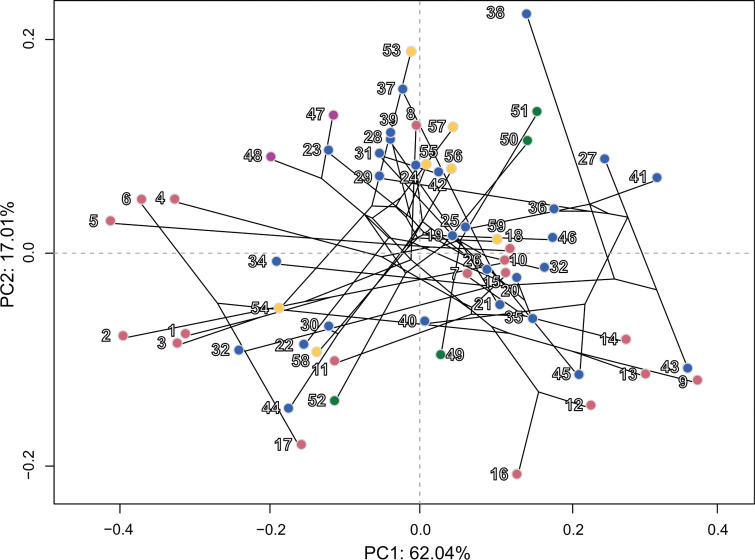
Phylomorphospace visualization. Phylomorphospace plot of principal components 1 and 2 incorporating the total-evidence phylogeny of the Lophioidei. Circle positions represent the average location in the morphospace for each genus. See S8 Table for genus-level labels. The color scheme for infraorders and families corresponds to Figs 1 and 2.

The consensus configuration of the relative warp analysis indicated the average body shape of the Lophioidei was an elongated compressiform with posteriorly located dorsal and anal fins located behind the midline of the length of the body, with the dorsal fin slightly larger than the length from the posterior end of the dorsal fin to the caudal peduncle, and an anal fin similar in length to the length from the posterior end of the anal fin to the caudal peduncle (Fig 3B). The dorsal fin is positioned posteriorly near the caudal peduncle and the anal fin is roughly half the length of the dorsal fin. The principal component analysis indicated that variation in the body shape among anglerfishes and allies (Figs 10A and 11), with principal components (PC) 1 and 2 describing 79.04% of the overall variation and principal components 3 and 4 describing 13.25% of the overall variation (S1 Fig) for a cumulative lateral body shape description of 92.31%. The first two principal components describe body-shape variation associated with the length and position of median fins and depth and elongation of body shape.

#### Body length.

The morphospace on the low score extremes on PC1 and PC2 represents a shorter body shape, while the high score extremes on PC1 and PC2 represents a longer body shape. The body length on the high score extreme on PC1 is ~1. 36 times greater than the low score extreme of PC1 and the body length on the high score extreme on PC2 is ~1. 18 times greater than the low score extreme of PC2. The body length of the low score of PC1 is ~1. 17 times greater than the low score extreme of PC2. The body length of the high score of PC2 is ~1. 02 times greater than the low score extreme of PC1.

#### Eye width.

The morphospace on the low score extremes on PC1 and PC2 represents a shorter eye diameter, while the high score extremes on PC1 and PC2 represents a wider eye diameter. The eye diameter on the high score extreme of PC1 is ~1. 39 times greater than the low score extreme of PC1, and the eye diameter on the high score extreme of PC2 is ~1. 22 times greater than the low score extreme of PC2.

#### Jaw length.

The morphospace on the low score extremes on PC1 and PC2 represents a shorter length from the uppermost point of the premaxilla to the posteriormost point of the jaw, while the high score extremes on PC1 and PC2 represents a longer length from the uppermost point of the premaxilla to the posteriormost point of the jaw. The length from the uppermost point of the premaxilla to the posteriormost point of the jaw on the high score extreme of PC1 is ~1. 38 times longer than the low score extreme of PC1, and the length from the uppermost point of the premaxilla to the posteriormost point of the jaw on the high score extreme of PC2 is ~1. 20 times greater than the low score extreme of PC2.

#### Median fins.

The morphospace on the low score extremes on PC1 and PC2 represents a shorter dorsal-fin length, while the high score extremes on PC1 and PC2 represents a longer dorsal-fin length. The dorsal-fin length on the high score extreme of PC1 is ~1. 49 times greater than the low score extreme of PC1, and the dorsal-fin length on the high score extreme of PC2 is ~1. 13 times greater than the low score extreme of PC2. The morphospace on the low score extremes on PC1 and PC2 represents a shorter anal-fin length, while the high score extremes on PC1 and PC2 represents a longer anal-fin length. The anal-fin length on the high score extreme of PC1 is ~1. 36 times greater than the low score extreme of PC1, and the anal-fin length on the high score extreme of PC2 is ~1. 19 times greater than the low score extreme of PC2.

#### Caudal peduncle.

The morphospace on the low score extremes on PC1 and PC2 represents a shorter and thinner caudal peduncle, while the high score extremes on PC1 and PC2 represents a longer and wider caudal peduncle. The caudal-peduncle thickness on the high score extreme of PC1 is ~1. 34 times greater than the low score extreme of PC1, and the caudal-peduncle thickness on the high score extreme of PC2 is ~1. 17 times greater than the low score extreme of PC2. The caudal-peduncle length on the high score extreme of PC1 is ~1. 35 times greater than the low score extreme of PC1, and the caudal-peduncle length on the high score extreme of PC2 is ~1. 28 times greater than the low score extreme of PC2.

Genera within the Antennarioideo were found to be distributed throughout the low and high score extremes of PC1 and PC2 (Fig 10A). The frogfishes had the highest disparity in body shape (Procrustes variance 0. 044) when compared to all the other lophioid infraorders and are found to be significantly different compared to the coffinfishes (P = 0. 048) and the monkfishes (P = 0. 033; Table 1). The frogfishes display a wide range of quantified body shapes across both the low and high scores of PC1 and PC2 (Fig 10A) that highly overlap the Ceratioideo, Lophioideo, and Ogcocephaloideo. Genera within the Chaunacoideo were recovered in a tight cluster in the low score of PC1 and the high score of PC2 (Fig 10A). The coffinfishes had the lowest disparity in body shape (Procrustes variance 0. 004) when compared to all the other lophioid infraorders and were found to be significantly different compared to the frogfishes (P = 0. 048) and the deep-sea anglerfishes (P = 0. 051; Table 1). The coffinfishes did not display a wide range of quantified body shapes and were restricted in their distribution on the principal component analysis. Genera within the Ceratioideo were distributed throughout the low and high score extremes of PC1 and PC2 (Fig 10A). The deep-sea anglerfishes had the second highest disparity in body shape (Procrustes variance 0. 042) when compared to all the lophioid infraorders and were found to be significantly different compared to the Lophioideo (P = 0. 034; Table 1) and only marginally different from the Chaunacoideo (Chaunacoideo [P = 0. 051; Table 1]). The deep-sea anglerfishes display a wide range of quantified body shapes across both the low and high scores of PC1 and PC2 (Fig 10A) that highly overlap the Antennarioideo, Lophioideo, and Ogcocephaloideo. Genera within the Lophioideo were distributed across the low and high scores of PC2 while typically centered on the middle of PC1 (Fig 10A). The monkfishes had second lowest disparity in body shape (Procrustes variance 0. 011) when compared to all the lophioid infraorders and were found to be significantly different compared to the frogfishes (P = 0. 033) and the deep-sea anglerfishes (P = 0. 034; Table 1) and were found to overlap the Antennarioideo, Ceratioideo, and, to a lesser extent, the Ogcocephaloideo. Genera within the Ogcocephaloideo were recovered to be minimally distributed across the low and high scores of PC1 and PC2, generally around the consensus point of the principal component analysis (Fig 10A). The batfishes had the third highest disparity in body shape (Procrustes variance 0. 031) when compared to all the lophioid infraorders and were not found to be significantly different than any other infraorder. The batfishes were found to overlap the Antennarioideo, Ceratioideo, and, slightly, the Lophioideo.

**Table 1:** Lophioid disparity values from the morphological disparity test by lophioid infraorders.

	Antennarioideo	Ceratioideo	Chaunacoideo	Lophioideo	Ogcocephaloideo
**Procrustes variance**	0.044	0.042	0.004	0.011	0.031
**P-Values**					
Antennarioideo	–	0.828	0.048*	0.033*	0.282
Ceratioideo		–	0.051	0.034*	0.337
Chaunacoideo			–	0.757	0.225
Lophioideo				–	0.270
Ogcocephaloideo					–

Asterisks denote significant values.

### Phylomorphospace

In most instances, closely related genera within infraorders tended to cluster nearer to each other in shape space with less closely related taxa being more morphologically disparate (Fig 11). For example, in some taxa of some deep-sea anglerfishes, similar body shapes of slightly globular bodies were found in Diceratiidae, Himantolophidae, and Melanocetidae. Still, in the deep-sea ceratioids, there are closely related lineages that have significantly different morphologies (e. g., *Borophryne*, *Caulophryne*, *Gigantactis*, *Himantolophus*, and *Thaumatichthys*.)

### Variation of body shape across marine habitats and depths

The relative warp and disparity analysis did not indicate a significant difference in quantitative body shape across different depth preferences and habitats (Fig 10B and Table 2). Across varying depths (0–200, 200–1,000, 1,000–3,000, 3,000–6,000 meters), there is high overlap across the principal component analysis (Fig 10B), with differences typically dominated by the higher sampled suborders (Antennarioideo and Ceratioideo). Depth categories of ≤ 200 and 200–1,000 m had the highest Procrustes variance (0. 042 and 0. 041) followed by 1,000–3,000 m (0. 029) and 3,000–6,000 m with a single genus sampling resulted in a Procrustes variance of 0. 000.

**Table 2:** Lophioid disparity values from the morphological disparity test by lophioid depth preference.

	≤ 200 m	200–1,000 m	1,000–3,000 m	3,000–6,000 m
**Procrustes variance**	0.042	0.041	0.029	0.000
**P-Values**				
≤ 200 m	–	0.861	0.165	0.095
200–1,000 m		–	0.206	0.103
1,000–3,000 m			–	0.251
3,000–6,000 m				–

No values were significant.

## Discussion

### Commentary on and examination of the synapomorphies for the Lophioidei

Several recent studies have listed synapomorphies for the Lophioidei (e. g., [102,146,148]). Our investigations indicate that four characters previously considered as synapomorphies by Pietsch and Arnold [102], Near and Thacker [146], and Betancur-R et al. [148] for the Lophioidei no longer provide evidence of this clade’s monophyly. These characters include (1) absence of the urohyal [16,149], (2) the pterygiophores of the first dorsal spines develop from a single condensation of tissue that separates into independent pterygiophores [91], (3) the first (the luring apparatus) and second dorsal spines are supported by the first pterygiophore [91], and (4) a single hypural plate formed by the fusion of the 2^nd^ ural centrum with 1^st^ preural centra that emanates from a single half centrum [10].

#### Urohyal.

Pietsch [16] noted the urohyal is absent in all taxa in the Lophioidei but did not explicitly list the character as a synapomorphy. In a later study by Datovo et al. [149], investigated the infrabrachial musculature of the Lophioidei and proposed the condition of the *rectus communis* and the absence of the urohyal as a synapomorphy for the Lophioidei. Their investigation produced no shared myological characters between the clade composed of the Lophioidei and Tetraodontoidei. At this time, we cannot comment on the condition of the *rectus communis* in members of the Lophioidei relative to members of the Tetraodontoidei or other outgroups; however, we note that Kusaka [97] identified and illustrated the presence of a urohyal in members of the Antennariidae, Chaunacidae, Lophiidae, and Ogcocephalidae. As such, this character might support the monophyly of the Ceratioideo, but it is not a synapomorphy for the Lophioidei.

#### Development of dorsal-spine pterygiophores.

Everly [91] (p. 415) investigated the larval stages of *Lophius americanus* and proposed “pterygiophores of the spinous dorsal fin develop from a single condensation of tissue that later divides or separates into independent pterygiophores. ” This character was proposed without comparative data. The acceptance of this synapomorphy without corroborative data across the non-lophiid lophioids combined with the lack of comparative data across acanthuriforms makes it premature to support this feature as a synapomorphy for the suborder. Further examination is needed.

#### First two dorsal spines supported by first pterygiophore.

Everly [91] (p. 415) investigated the larval stages of *Lophius americanus* and proposed that the first (the luring apparatus) and second dorsal-fin spines are supported by the first pterygiophore as a synapomorphy for the Lophioidei, assuming a batrachoidiform sister group. By examining specimens and the literature, we found evidence that all of the included outgroup genera in our total-evidence phylogeny had a first pterygiophore that supports both the first and second dorsal-fin spines [95,109,110]. Though not a synapomorphy for the Lophioidei, it does raise the question as to whether this character is restricted to either within or beyond the larger acanthuriform radiation. Recent percomorph phylogenies (e. g., [30,35,145] combined with the comparative anatomical data of Johnson [95] and similar studies suggest that the complex evolutionary history of this character will likely support relationships within this group.

#### Complex caudal-fin elements.

This combination of characters (a single hypural plate formed by the fusion of the 2^nd^ ural centrum with 1^st^ preural centrum that emanates from a single half centrum) was first proposed by Rosen and Patterson [10] as a synapomorphy for our Lophioidei. Subsequent authors have variously noted this feature as a synapomorphy for the Lophioidei [1,16,102,146,148]. Given the variation in caudal skeleton element fusions seen across fishes [92], this complex series of features is best examined in terms of independent fusions of the hypurals, parahypurals, and vertebral elements. We performed a preliminary coding and optimization of these characters against our tree with the following characters: Parahypural and hypural one: (0) separate, (1) fused or one element lost; hypurals one and two: (0 separate), (1) fused or one element lost; hypurals three and four: (0) separate, (1) fused or one element lost; hypurals four and five: (0) separate, (1) fused or one element lost; upper and lower elements of caudal-fin: (0) separated, (1) fused; epurals: (0) absent, (1) present; epural counts: (0) three epurals, (1) two or one epurals; epural counts: (0) two or three epurals, (1) one epural. When optimized individually, none of these independent caudal fusions were found to support the monophyly of the Lophioidei or the Lophioidei + Tetraodontoidei [66,92,111].

### Morphological evidence for the interrelationships and monophyly of the Lophioidei

Our total-evidence analysis inferred a monophyletic Lophioidei (Fig 6) that was consistent with recent molecular phylogenetic studies (e. g., [30,34,36,38–40,145]. Our recovery of a sister-group relationship between the Lophioidei and the Tetraodontoidei was also found by [26,27,31–35,40,145].

Among our outgroup taxa, we recovered Antigoniidae sister to Tetraodontoidei+Lophioidei. The sister group to that clade was Priacanthidae+Caproidae. Before molecular analyses, the Antigoniidae, Caproidae, and Priacanthidae have had an uncertain placement, being classified variously among percomorph or zeiform allies (e. g., [23,95,107,113]. They are generally now recognized in the Acanthuriformes, near the Lophioidei and Tetraodontoidei [25,30,33,65,145]. In the following two sections we will discuss and list the relevant synapomorphies for the listed clades recovered by our analysis.

### Antigoniidae + Lophioidei + Tetraodontoidei

#### Character 89.

Number of caudal-fin rays (Fig 7). Tyler and Sorbini [83] noted that the Tetraodontoidei are united by having 12 or fewer principal caudal-fin rays. Given the sister-group relationship we recovered between the Lophioidei and the Tetraodontoidei, we examined the distribution of this character among the Lophioidei and outgroup acanthuriforms. Instead of only the Tetraodontoidei, we found this character unites a clade consisting of *Antigonia*, Lophioidei, and Tetraodontoidei (state = 1) [17,83,113]. In contrast, the remaining acanthuriform outgroups have 14 or more principal caudal-fin rays (state = 0) [83,95,107–110,113].

### Lophioidei + Tetraodontoidei

#### Character 90.

Condition of gill opening (Fig 7). Early on Rafinesque [4], used the restricted gill openings near the base of the pectoral fin without the coverage of an operculum to group the modern Balistidae and Lophiidae into his Balistini. Subsequent authors have highlighted the presence of a restricted gill opening for the Lophioidei or the Tetraodontoidei (e. g., [16,17,83,98,107,108], with recent authors explicitly supporting the monophyly of either the Lophioidei or Tetraodontoidei with the presence of a restricted gill opening even when treating them as a sister group [1,102,146,148,150]. Given the sister-group relationship between these two suborders, we group the Lophioidei and Tetraodontoidei together by the shared presence of a reduced gill opening (state = 1). In contrast, the remaining acanthuriform outgroups have an operculum covering their larger gill openings (state = 0) [95,98,107–110,113,150].

#### Character 91.

Occurrence of anal spines (Fig 7). Tyler [66] described the number of anal-fin rays throughout the Tetraodontoidei without explicitly mentioning the lack of anal-fin spines throughout the suborder. Tyler and Sorbini [83] would later note that the Tetraodontoidei are united by a lack of anal-fin spines when zeiforms and beryciforms were considered allies. Subsequent authors have variously noted this feature as a synapomorphy for the Tetraodontoidei (e. g., [1,146,148,150]. Instead of only the Tetraodontoidei, we found this character unites the Lophioidei and the Tetraodontoidei (state = 0). In contrast, the remaining acanthuriform outgroups have anal-fin spines present (state = 1) [95,107–110,113].

#### Character 92.

Occurrence of nasals (Fig 7). Tyler and Sorbini [83] noted the absence of nasals in the Tetraodontoidei and used it as a synapomorphy for the clade. Subsequent authors have used this feature as a potential synapomorphy for the Tetraodontoidei (e. g., [1,146,148,150]. Pietsch [16] noted that the absence of nasals is a synapomorphy for the Lophioidei. Instead of nasals being absent independently in only the Lophioidei or the Tetraodontoidei, we found this character unites these two suborders (state = 0). In contrast, the remaining acanthuriform outgroups have nasals present (state = 1) [83,107–110,113].

#### Character 93.

Occurrence of the basisphenoid (Fig 7). Tyler and Sorbini [83] provided commentary regarding the absence of the basisphenoid in most tetraodontoid families, noting its presence in molids and triodontids, but they did not explicitly list this state of the basisphenoid as a synapomorphy for the Tetraodontoidei due to its uncertain optimization when optimized with the Zeiformes. Pietsch [16] noted that all lophioids lack a basisphenoid but did not list this state as a synapomorphy for the Lophioidei or comment on the presence of the basisphenoid in the Batrachoididae [151]. Though not explicitly noted as a synapomorphy for either the Lophioidei or Tetraodontoidei, we found this character unites the Lophioidei and the Tetraodontoidei (state = 0). In contrast, the remaining acanthuriform outgroups except for *Siganus*, lack a basisphenoid (state = 1) [83,95,107–110,113].

#### Character 94.

Occurrence of ribs (Fig 7). Tyler and Sorbini [83] noted the absence of ribs in the Tetraodontoidei and suggested this feature united the clade. Regan [7] noted the absence of ribs as a shared character for the Pediculati (Batrachoididae + Lophioidei) with subsequent authors variously noting this feature as a synapomorphy for the Lophioidei [1,137,146]. Instead of ribs being absent independently in only the Lophioidei or the Tetraodontoidei, we found this character unites these two suborders (state = 0). In contrast, the remaining acanthuriform outgroups have ribs (state = 1) [83,95,107–110,113].

#### Character 95.

Presence of a modified dorsal-fin spine used as a luring apparatus (Fig 7). The luring apparatus is several characters, including modifications and specializations to the dorsal-fin spines, cranial bones, and the associated muscles and innervations [7,16,18,102]. We found that this character continues to unite the Lophioidei (state = 1). In contrast, the remaining acanthuriform and tetraodontoid outgroups do not have a modified dorsal-fin spine used as a luring apparatus (state = 0) [83,95,107–110,113].

#### Character 96.

Condition of the oral teeth (Fig 7). Fink [84] first described tooth attachment in lophioids and identified tooth attachments that facilitated teeth that could hinge in a posterior axis of rotation (type 4) in the Ceratiidae, Ogcocephalidae, and Oneirodidae. In our examined material from all lophioid suborders, we found highly depressible oral teeth; whereas the tetraodontoids have non-depressible teeth that can range from beak-like to molariform or fused plate-like teeth (A. J. M. pers. obs.; [66]). We found that this depressible condition of the oral teeth unites the Lophioidei (state = 1). In contrast, we found that our remaining acanthuriform outgroups have non-depressible teeth (A. J. M pers. obs.; state = 0; [66,84]. While not comparatively examined across other acanthuriforms, we note that the pharyngeal teeth are hinged in *Lophius* (A. J. M pers. obs.)

#### Character 97.

Shape of egg mass (Fig 7). Several studies [85,89,152] have investigated the egg masses produced by lophioids, describing them as gelatinous, scroll-like sheaths. Subsequent authors have variously suggested that this feature may be a synapomorphy for the Lophioidei [1,16,18,102,146]. We found that this character continues to unite the Lophioidei (state = 1). In contrast, the remaining acanthuriform outgroups produce eggs with various morphological adaptations that are not enveloped in this gelatinous sheath (state = 0) [77,85,86,89–91,98,101,102,104,108,150–152].

#### Character 98.

Condition of the epiotics in relation to the parietals and supraoccipital (Fig 7). Regan [7] first noted the separation of the epiotics from the parietals that meet medially posterior to the supraoccipital in the Lophioidei. Subsequent authors have variously noted this feature as a potential synapomorphy for the Lophioidei (e. g., [1,16,18,102,104,146]. Tyler and Sorbini [83] noted the absence of the parietal in the Tetraodontoidei. We support the earlier assertion that this character unites the Lophioidei (state = 1) because the epiotics in remaining acanthuriform outgroups either make contact with the parietals [108,109,113] or lack a parietal in the tetraodontoids [110] (state = 0).

#### Character 99.

Length and shape of the pectoral radials (Fig 7). Length of the pectoral radials (Fig 7). The elongation of the pectoral radials in lophioids was first described by Cuvier [5]. This observation led to the classification of the “Acanthoptérygiens à pectorales pédiculées,” which eventually became the Pediculati and was composed of the Batrachoididae and Lophioidei. Subsequent authors used elongate pectoral radials to unite the Batrachoididae and Lophioidei [7,9,11,15] or to unite only the Lophioidei [1,16,18,102,146]. We agree with these authors that elongate pectoral radials diagnose the Lophioidei (state = 1). In contrast, the remaining acanthuriform outgroups have more typical square or hour-glass-shaped pectoral radials (state = 0) [83,95,107–110,113].

#### Other lophioid characters.

Several authors have identified characters that may indicate shared ancestry between lophioids and other acanthuriforms that we were not able to broadly examine for this study. Many contributions [17,98,99,103] in the Ahlstrom Symposium independently describe, with varying interpretations, the presence of a prominent, inflated, transparent structure encompassing certain larvae of the Lophioidei and Tetraodontoidei. Pietsch [17] described the larvae of some antennariids, ceratioids, chaunacids, lophiids, and ogcocephalids as having highly inflated skin around the body. Additional studies [43,103] described ceratioid larvae as having a “inflated transparent skin” and “balloon-like envelope.” In the Tetraodontoidei, Leis [98] and Aboussouan [99] describe the occurrence of a “vesicular dermal sac” found in the Diodontidae, Molidae, Ostraciidae, Tetraodontiidae, and briefly in the Balistidae. This condition was not found in the Monocanthidae, Triacanthidae, and Triacanthodidae. Later Baldwin [153], investigated color patterns of marine teleost larvae, examining a larval ostraciid and an unidentified lophioid. The study found that the prominent, inflated, transparent structures encompassing the larvae are covered with xanthophores, suggesting this may be a shared character between the Lophioidei and Tetraodontoidei. It was also noted by Baldwin [153] that within the Tetraodontoidei, the prominent, inflated, transparent structures are restricted to the preflexion stage, although the same condition persists after the flexion stage.

Nakae and Sasaki [154] identified a shared accessory lateral line with three accessory neuromasts with a newly reconstructed preopercular line resulting from free space caused by the restricted gill openings between the Tetraodontoidei and the Lophiidae. Chanet et al. [155] focused their investigation on identifying soft-tissue characters that may unite the Lophioidei and Tetraodontoidei. They suggested that rounded and anteriorly disposed kidneys, a compact thyroid nested in a thyroidian sinus [156], an abbreviated spinal cord, and clusters of supramedullary neurons in the rostral part of the spinal cord may unite these two groups. Finally Farina and Bemis [157], noted that the path of the adductor hyohyoideus that dorsally extends through the branchiostegal rays, the ventromedial wall of the gill opening, to the dorsal midline of the body resembles that of the Tetraodontoidei, and they suggested it may be a synapomorphy of the Lophioidei and Tetraodontoidei. Slow breathing or “holding breath” behavior hypothesized to serve in reducing metabolic rate, reducing energetic demands, or as a defense mechanism has also been observed in the monkfishes, coffinfishes, and frogfishes [102,140,157], and these have been suggested to be similar in ability to the behaviors of some tetraodontoids to inhale water to inflate [140,157].

### Morphological evidence and commentary on the intrarelationships of the Lophioidei

#### Lophioideo.

**Fig 12:**
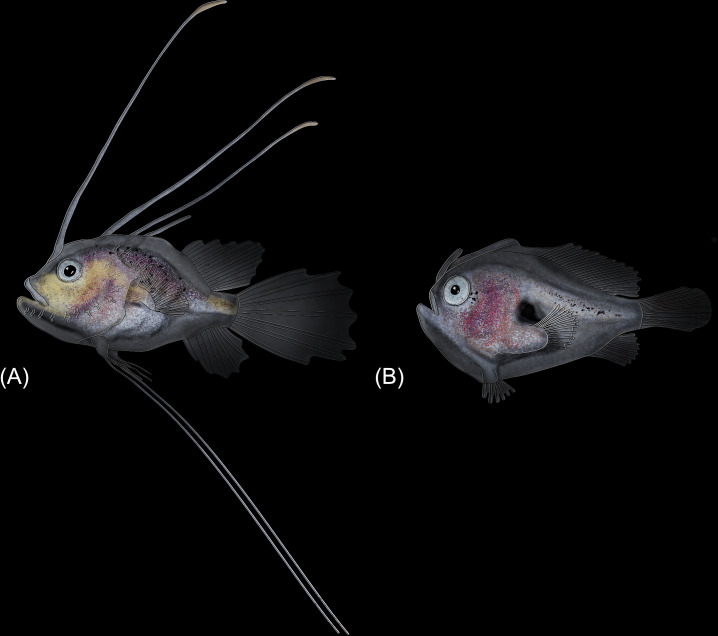
Elongated and non-elongated fin elements in lophioid larvae. Illustrations highlighting the derived and ancestral states of dorsal- and pelvic-fin rays among lophioid infraorders: (A) *Lophiodes* (Lophioideo), elongated fin elements present; (B) *Histrio* (Antennarioideo), elongated fin elements absent. Fish illustrations by Alex Maile.

***Character 100:*** Length of fin elements of the dorsal and pelvic fins in larvae (Figs 7 and 12). In the Lophioideo (monkfishes and goosefishes), the larvae possess elongate fin elements associated with the dorsal and pelvic fins (Fig 12). This character has been noted in various genera by several authors [17,82,91,158]. Although it has never been suggested as a synapomorphy for the infraorder, we found that this character is found in all lophioid genera and should be treated as a synapomorphy. In contrast, the non-lophiid lophioids, tetraodontoids, antigoniids, caproids, priacanthids, and siganids do not have both elongate dorsal- and pelvic-fin elements [17,90,91,93,94,96,100,104–106,112]. Our total-evidence results only recognized this one new feature as a synapomorphy (Fig 7; S4 Table).

The Lophioideo is inferred to be monophyletic and the sister to all other infraorders of the Lophioidei in all three analyses (Figs 4–6). This monophyly and relationship is consistent with most prior morphological, molecular, and combined studies [16,19,34,36–40,42] but Shedlock et al. [41] recovered *Lophius* in a more apical placement in the tree. All lophiid genera represented by multiple species in our analyses were found to be monophyletic. Caruso [159] did not report any synapomorphies for the Lophiidae, but he noted the presence of a single anterior articular spine as a synapomorphy for a clade composed of *Lophiodes*, *Lophiomus,* and *Lophius*. Carnevale and Pietsch [160] identified eight characters without homoplasy to support the monophyly of the Lophiidae when their analysis included only two antennarioid outgroups: (1) mesethmoid absent; (2) ascending process of the premaxilla autogenous; (3) ectopterygoid and endopterygoid fused; (4) anterodorsal process of subopercle prominent, articulating through connective tissue with the anteroventral margin of opercle; (5) teeth on fifth ceratobranchial restricted to discrete rows along the lateral and medial margins; (6) eight caudal-fin rays; (7) cleithral spine present; (8) skin naked. We did not investigate these characters in the Ceratioideo, Chaunacoideo, or Ogcocephaloideo. That said, we note that Betancur-R et al. [148] pointed readers to Pietsch [16] for synapomorphies for the family, but that study did not include any synapomorphies for the Lophiidae.

#### Antennarioideo.

The reclassified Antennariidae (Antennarioideo) is supported by two morphological synapomorphies ([19]; Fig 7; S4 Table): (1) the interhyal with a medial, posterolaterally directed process that meets the respective preopercle [16]; (2) the illicial pterygiophore and pterygiophore of the third dorsal-fin spine with highly compressed, blade-like dorsal expansions [16]. We recovered a monophyletic Antennarioideo in all three analyses (Figs 4–6) with taxonomic coverage in our molecular and combined analyses that includes of 19 out of 23 genera and all subfamilies. The monophyly of this clade is consistent with prior morphological, molecular, and combined studies [16,19,34,36–40,74] but differed from Shedlock et al. [41] who recovered a paraphyletic Antennarioideo.

Arnold and Pietsch [74] described Histiophryninae and Hart et al. [38] described Tathicarpidae and Rhycheridae. Based on the results of our total-evidence and molecular analyses, we recommended recognizing an Antennariidae that is equivalent to the Antennarioidei, so we recognize these previously described families [2] as subfamilies and described Fowlerichthyinae. We note that Hart et al. [38] provided a description of Histiophrynidae; yet this family-group name was already described by Arnold and Pietsch [74]. The composition of our Histiophryninae and Rhycherinae varies across studies [2,38,40,74], so we emphasize that we are recognizing these as *Lophiocharon* for Lophichthyinae, *Histiophryne* for Histiophryninae, and *Allenichthys*, *Echinophryne*, *Kuiterichthys*, *Phyllophryne*, *Porophryne*, and *Rhycherus* for Rhycherinae. That said, our placement of *Allenichthys*, should be considered tentative because it has not been included in any phylogenetic analyses, but we place it in Rhycherinae following the recommendation of Hart et al. [38].

Hart et al. [38], Brownstein et al. [39], and Miller et al. [40] recovered an Antennariinae, which included our Antennariinae and Fowlerichthyinae as the sister group of their Brachionichthyinae, Histiophryninae, Rhycherinae, Tathicarpinae, and Tetrabrachiinae (Fig 2). We recovered similar relationships. Hart et al. [38], Brownstein et al. [39], and Miller et al. [40] did not include the Lophichthyinae, which we recovered sister to the Tathicarpinae. Tetrabrachiinae was found as sister group to the Histiophryninae and Tathicarpinae by Hart et al. [38] and Brownstein et al. [39], as sister group to Tathicarpinae by Miller et al. [40], and we instead recovered Lophichthyinae + Tathicarpinae as sister group to the Histiophryninae and Tetrabrachiinae. We also recovered the same sister-group relationship between the Brachionichthyinae and Rhycherinae that was found by Hart et al. [38], Brownstein et al. [39], and Miller et al. [40]. Brownstein et al. [39], following Near and Thacker [146], also treated the Antennarioideo as a single family.

#### Ogcocephaloideo.

The Ogcocephaloideo is supported by four morphological synapomorphies [37]; Fig 7; S4 Table): absence of the first epibrachial (43) convergent in the Ceratioideo; a tiny opening of the escal pore leading from the central cavity to the outside (57); third cephalic dorsal-fin spine and pterygiophore absent (60); posttemporal fused to the neurocranium (63). The Ogcocephaloideo was found monophyletic in all three analyses (Figs 4–6), which is consistent with prior morphological, molecular, and combined studies [16,19,34,36–39,42]. All genera with multiple species included in our analyses were found to be monophyletic. The monophyly of the Ogcocephaloideo was discussed by Bradbury [87], and she proposed several characters to diagnose the family: (1) glandular and distinct shape of the esca; (2) illicium in a resting position rests in an illicial cavity that opens on the anterior face of the “forehead”; (3) well-developed scales. Nagareda and Shenker [139,161], after Bradbury [87] found evidence that the glandular escae found in ogcocephalids produce chemicals for prey attraction. The clade consisting of the Antennaroideo and the Ogcocephaloideo is recovered as the sister group to the clade composed of the Chaunacoideo and the Ceratioideo (Figs 4–6). Regan’s [7] classification indicated a close relationship among the Antennarioideo, Chaunacoideo, and Ogcocephaloideo within his Antennariiformes. Later Pietsch [16], separated the Antennariidae from a clade composed of the Chaunacidae + Ogcocephalidae, but he treated them as the suborder Antennarioidei and provided four characters for the Chaunacidae + Ogcocephalidae with four morphological characters: (1) posteriormost branchiostegal ray exceptionally large; (2) gill teeth tiny, arranged in a tight cluster at apex of pedicel-like tooth plates; (3) gill filaments of first gill arch absent; (4) illicial bone, when retracted, lying within an illicial cavity.

#### Chaunacoideo.

The Chaunacoideo is supported by one morphological synapomorphy ([19]; Fig 7; S4 Table): presence of an expanded and squared off posteroventral margin of the articular (35). The Chaunacoideo was monophyletic in all three analyses (Figs 4–6). A monophyletic Chaunacoideo is consistent with prior morphological, molecular, and combined studies [16,19,34,36–39,75]. All genera with multiple species represented in our analyses were found to be monophyletic.

#### Ceratioideo.

The Ceratioideo was supported by 17 morphological synapomorphies ([19]; Fig 7; S4 Table): palatine reduced and toothless (17); postmaxillary process of the premaxilla absent (28); thick anterior-maxillomandibular ligament highly reduced or absent (30); basihyal absent (38); first epibranchial simple without any ligamentous connection to the second epibranchial (43); epurals absent (50); innermost four caudal-fin rays bifurcated (53); escae enclose an expanded central cavity containing bioluminescent bacteria (56); tiny opening leading from the central cavity to the outside of the esca present (57); third cephalic dorsal-fin spine and pterygiophore absent (60); rays of the dorsal and anal fins comparatively long (63); pelvic fins absent in both juveniles and adults of both sexes (67); metamorphosed males of all ceratioids only a small fraction of the size of females (72); olfactory organs of males large, reaching between 12. 5–21. 7% of head length (75); posterior nostrils directed laterally and anterior nostrils directed anteriorly (79); sexual dimorphism of the illicial apparatus present (86); and pelvic-fin rays absent in larvae (88).

The Ceratioideo was recovered as sister to the Chaunacoideo in all three analyses; the UCE-only analysis (Fig 4) included seven genera, the molecular analysis (Fig 5) included 24 genera, and the total-evidence analysis (Fig 6) included 31 out of 35 genera. The resolution of the Ceratioideo that encompasses all previously recognized deep-sea anglerfish families recovered relationships that were different from previous studies [19,34,36–40]. We recovered the Caulophrynidae as the sister group of all other ceratioid anglerfishes. Hart et al. [38] did not recover this relationship and instead found the Melanocetidae as the sister to all other ceratioid anglerfishes. Because this relationship differed from previous relationships, we downloaded several UCEs from GenBank [123] produced by Hart et al. [38] to confirm their sequence IDs using comparisons with mitochondrial sequence databases (BOLD [124] and GenBank [123]). Their *Caulophryne jordani*, *Caulophryne pelagica*, *Melanocetus johnsonii*, and *Melanocetus murrayi* were identified as *Melanocetus murrayi*, *Melanocetus johnsonii*, *Caulophryne jordani*, and *Caulophryne jordani*, respectively (Fig 2. See asterisks). This misidentification was also discovered by Miller et al. [40] and due to potential misidentifications, we largely excluded SRAs from Hart et al. [38] in our analysis, though we incorporated one taxon (*Ogcocephalus radiatus* identified as *Ogcocephalus cubifrons*) as a test case. The SRAs from Brownstein et al. [39] were made available after completion of our analyses, so they were not incorporated. With this taxonomic correction in Hart et al. [38], our analyses support Caulophrynidae as the sister to all other ceratioid anglerfishes and Himantolophidae as sister to Melanocetidae. Brownstein et al. [39] recovered Gigantactinidae as the sister group of the Centrophrynidae, Ceratiidae, Diceratiidae, Himantolophidae, Linophrynidae, Melanocetidae, Neoceratiidae, Oneirodidae, and Thaumatichthyidae while Miller et al. [40] recovered the Gigantactinidae as sister to the Linophrynidae. Brownstein et al. [39] recovered Neoceratiidae as the sister group of Linophrynidae and Ceratiidae as sister to Centrophrynidae, Diceratiidae, Himantolophidae, Melanocetidae, Oneirodidae, and Thaumatichthyidae while Miler et al. [40] recovered Thaumatichthyidae as sister to Neoceratiidae. Brownstein et al. [39] recovered Centrophrynidae as the sister group to a clade consisting of Diceratiidae, Himantolophidae, Melanocetidae, Oneirodidae, and Thaumatichthyidae while Miller et al. [40] recovered Centrophrynidae as the sister group to Ceratiidae, Gigantactinidae, Linophrynidae, Neoceratiidae, Thaumatichthyidae, Diceratiidae, Melanocetidae, Himantolophidae, and Oneirodidae. *Lasiognathus* was recovered among the oneirodids rather than as a member of the Thaumatichthyidae [19] as was shown by previous studies [37,39,40,75]; this genus should be classified in the Oneirodidae.

### Evolutionary patterns of body shape, depth, and habitat transitions

There is significant quantitative evidence of body-shape changes within the Lophioidei. We find distinct clustering of body-shape patterns among infraorders but not associated with variation in depth occurrences (Fig 10) as was also found by Miller et al. [40]. To observe depth and habitat transitions, a conservative method was employed to average these occurrences across lophioid taxa. However, it should be noted that certain taxa may span a broader depth range beyond our designated limits. Additionally, behaviors such as ontogenetic changes in vertical migrations observed in ceratioids [104] are not fully accounted for using our ancestral character-state coding, and our depth values were conservatively averaged. The ancestral character-state reconstructions (Figs 8 and 9) indicate that the common ancestor of the Lophioidei was benthic/demersal at depths of 200–1,000 m, with several upward transitions to shallower depths and several downward transitions to deeper waters. The depth ranges of ≤ 200 m and 1,000–3,000 m are the most genus rich among the lophioids and are dominated by the two most variable body-shape infraorders (Antennarioideo and Ceratioideo), along with the significant overlap of body shapes at other depths; this accounts for the absence of significant differences in body shapes across various depths. Our results align with the findings of Derouen et al. [42] regarding the depth transitions in Ogcocephaloideo. Additionally, our study encompasses a broader range of genera, offering a more comprehensive insight into to the depth transitions undertaken by the Lophioidei.

The two large body-shape clusters, the Antennarioideo and the Ceratioideo, generally cover a similar morphometric spread across principal components 1 and 2 (Fig 10). Though the spread of these body shapes is similar, the two infraorders were found in separate depths and habitats, yet their body disparity values are similar and were not found to be significantly different from one another. Though not a major overlap Miller et al. [40], did recover a slight overlap in body shape shape clusters between the Antennarioideo and the Ceratioideo. In their investigation of body-shape changes across 3,000 marine teleost species, Martinez et al. [162] found that the body-shape disparity nearly doubled from inshore/shallow depths to deep-sea depths and suggested that body-shape evolution trended toward shapes that facilitated slow and periodic swimming. The ceratioid anglerfish adult females have typically been found drifting in the water current [135,138]. Their large heads, poorly ossified skeletons, and weakly developed muscles do not facilitate fast and frequent swimming [104], yet the batfishes [134], coffinfishes [75], frogfishes [102], and monkfishes [157] all exhibit slow and periodic swimming and are predominantly sit and wait predators. With varying body-shape disparities across our analysis, the similar disparity values found in the Antennarioideo and Ceratioideo are unlikely to be explained by the specialization of their respective locomotion. The greater variation in body shapes within these two infraorders may have evolved to facilitate a particular predation technique, possibly by enhancing the motion or attractiveness of the luring apparatus.

The body plans of dorsoventrally flattened monkfishes found predominantly on soft bottom and continental shelves (200–1,000 m) and batfishes found predominantly in benthic/demersal midwater depths (0–1,000 m) may be suitable for attracting benthic/demersal prey, but frogfishes and deep-sea anglerfishes may require changes and larger variation in their body shape to facilitate prey taken in their respective habitats (≤ 200 m and 1,000–3,000 m). A consequence of 2D geometric morphometrics, as performed in this analysis, is the loss of information regarding the change in body shape from a lateral depth perspective, as the frogfishes are generally narrower compared to the deep-sea anglerfishes [102,104]. Approaching the Lophioidei with a 3D geometric method may provide more informative perspective regarding the changes in overall body shape across depth and habitat changes in this group [40,163]. Our study provides two molecular and one total-evidence phylogenies for the Lophioidei highlighting the depth and habitat transitions and quantifying the body-shape changes undergone by the suborder through genus-level coverage.

## Supporting information

S1 Fig Geometric morphometric principal component analysis 3 + 4. (TIFF)

S1 Table Material examined. (DOCX)

S2 Table References for images of Lophioidei representatives used that were not photographed by the authors. (XLSX)

S3 Table Sequence voucher and accession information used in phylogenetic analysis (see Figs 4–6). Mitochondrial data extracted from UCE extraction are not listed.
(XLSX)

S4 Table Morphological matrix. Characters 1–88 from Pietsch and Orr (2007). Characters 89–100 described in discussion.
(XLSX)

S5 Table Partitioning scheme for total evidence analysis. (DOCX)

S6 Table Expanded molecular (UCE + mitochondrial data) matrix. (NEX)

S7 Table Habitat and depth character matrix. (XLSX)

S8 Table Genus labels associated with geometric morphometric analysis (Figs 10 and 11). (XLSX)
