# Supplementary figures and images for: A total-evidence phylogenetic approach to understanding the evolution, depth transitions, and body-shape changes in the anglerfishes and allies (Acanthuriformes: Lophioidei)
Source: PLoS One. 2025 May 2;20(5):e0322369. doi: 10.1371/journal.pone.0322369 (PMC12047784; doi:10.1371/journal.pone.0322369)

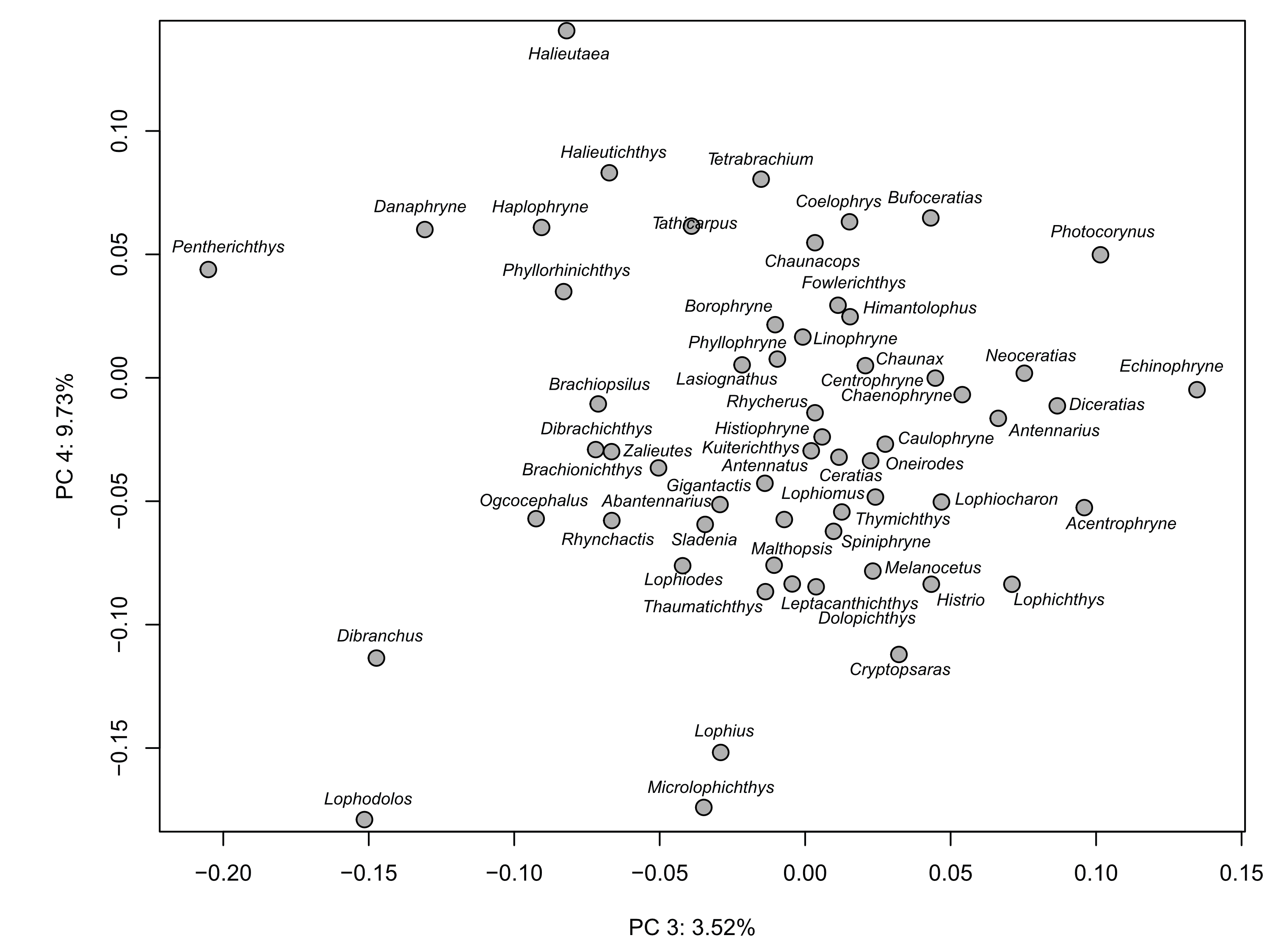

Supplement: S1 Fig — (TIFF) [file pone.0322369.s001.tiff]
