# Supplementary material for: A total-evidence phylogenetic approach to understanding the evolution, depth transitions, and body-shape changes in the anglerfishes and allies (Acanthuriformes: Lophioidei)
Source: PLoS One. 2025 May 2;20(5):e0322369. doi: 10.1371/journal.pone.0322369 (PMC12047784; doi:10.1371/journal.pone.0322369)
Supplement: S1 Table — (DOCX) [file pone.0322369.s002.docx]

**S1 Table. Material examined.**

**Geometric morphometric material examined.** Formalin-fixed lophioid specimens preserved in 75% ethanol examined and digitized for geometric morphometric analyses: *Abantennarius coccineus*, LACM 9383-2; *Antennatus* *strigatus*, LACM 20677; Borophryne *apogon*, LACM 30053-10; *Bufoceratias* *wedli*, LACM 34272, LACM 57232-1; *Caulophryne* *polynema*, LACM 33923-1; *Centrophryne* *spinulosa*, LACM 31105-24; *Ceratias* *tentaculatus*, LACM 11025-7; *Chaenophryne* *melanorhabdus*, LACM 9810-21; *Chaunax* *sp*., LACM 44750-3; *Cryptopsaras* *couesii*, LACM 11231-1; *Dibranchus atlanticus* FMNH 65256; *Dolopichthys* *longicornis*, LACM 9612-49; *Gigantactis* *gargantua*, LACM 30996-16; *Gigantactis* *vanhoeffeni*, LACM 45001-1; *Haplophryne* *mollis*, LACM 11235-25; *Himantolophus* *albinares*, LACM 57239-2; *Himantolophus* *cornifer*, LACM 33325-1; *Himantolophus* *sagamius*, LACM 43760-1, LACM 60082-1; *Histrio* *histrio*, LACM 8975-1; *Kuiterichthys* *sp*., LACM 11537-1; *Leptacanthichthys* *gracilispinis*, LACM 33625-2; *Linophryne arborifera,* FMNH 49639; *Linophryne* *coronata*, LACM 9254-34; *Linophryne* *densiramus*, LACM 38440-1; *Linophryne* *indica*, LACM 36046-11; *Lophiocharon* *trisignatus*, LACM 54171-1; *Lophiomus setigerus* FMNH 121120; LACM 44743-7; *Melanocetus* *johnsonii*, FMNH 121606, LACM 57236-2; *Melanocetus* *murrayi*, LACM 36113-1; *Microlophichthys* *microlophus*, LACM 33607-1; *Ogcocephalus cubifrons* FMNH 38584; *Oneirodes* *acanthias*, LACM 9100-1; *Oneirodes* *eschrichtii*, LACM 6697-5; *Oneirodes* *luetkeni*, LACM 33628-1; *Oneirodes* *notius*, LACM 10716-6; *Phyllorhinichthys* *micractis*, LACM 57238-7; *Rhynchactis leptonema* FMNH 124824; *Spiniphryne* *gladisfenae*, LACM 10970-2; *Zalieutes* *elater,* FMNH 89523.

**Morphological character material examined.** Additional formalin-fixed whole or cleared and stained specimens (C&S) examined for morphological characters not used in geometric morphometric analysis: *Antigonia* capros, FMNH 121028; KUI 27136; KUI 42893; KUI 42760 (C&S), *Acanthaluteres vittiger*, FMNH 135887; *Alutera monoceros*, FMNH 66376; *Anacanthus barbatus*, FMNH 45820; *Antennarius multiocellatus,* KUI 23874; *Arothron nigropunctatus*, FMNH 123403; *Balistes capriscus*, KUI 42812 (C&S); Balistoides *conspicillum*, FMNH 127479; *Canthidermis maculata*, FMNH 89271; *Cantherhines dumerilii*, FMNH 146474; *Canthigaster rostrata*, FMNH 48473; *Capros aper,* USNM 320065; *Chaunax pictus*, MCZ 166074; *Chelonodon patoca*, FMNH 40676; *Chilomycterus schoepfi*, FMNH 113489; *Colomesus psittacus*, FMNH 91869; *Diodon liturosus*, FMNH 127832; *Guentheridia formosa*, FMNH 90236; *Haemulon sciurus* AMNH 28960SW; *Halieutichthys* *aculeatus*, KUI 14001 (C&S) *Hollardia hollardi*, FMNH 59997; *Lactophrys bicaudalis*, FMNH 59862; *Leiognathus equulus*, FMNH 51645; *Lophiomus setigerus,* KUI 41736; *Macrorhamphosodes uradoi*, FMNH 121217; *Melichthys vidua*, FMNH 124013; *Monacanthus chinensis*, FMNH 110930; *Morone saxatilis,* AMNH 229546; *Odonus niger*, FMNH 118793; *Oxymonacanthus longirostris*, FMNH 90728; *Ostracion cornutus*, FMNH 47547; *Ostracion cubicus*, FMNH 119560; *Parahollardia lineata*, FMNH 46687; *Pervagor melanocephalus*, FMNH 127632; *Porichthys plectrodon*, KUI 27037 (C&S), *Priacanthus arenatus*, KUI 27012; *Pseudaluteres nasicornis*, FMNH 76689; *Pseudobalistes flavimarginatus*, FMNH 138883; *Rhineacanthus aculeatus*, FMNH 120081; *Siganus stellatus*, FMNH 138863; *Sphoeroides tyleri*, FMNH 87103; *Stephanolepis hispidus*, FMNH 66339; *Sufflamen albicaudatum*, FMNH 143381; *Takifugu stictonotus*, FMNH 141441; *Tetraodon mbu*, FMNH 128026; *Tetrosomus reipublicae*, FMNH 121211; *Torguigener pleurogramma*, FMNH 21735; *Triacanthus brevirostris*, FMNH 57424; *Xanthichthys auromarginatus*, FMNH 118800; *Zebrasoma flavescens* KUI 18217.
