# Supplementary material for: A total-evidence phylogenetic approach to understanding the evolution, depth transitions, and body-shape changes in the anglerfishes and allies (Acanthuriformes: Lophioidei)
Source: PLoS One. 2025 May 2;20(5):e0322369. doi: 10.1371/journal.pone.0322369 (PMC12047784; doi:10.1371/journal.pone.0322369)
Supplement: S5 Table — (DOCX) [file pone.0322369.s006.docx]

**S5 Table. Partitioning scheme for total evidence analysis.**

#nexus

begin sets;

charset Morph = morphtotal.phy:MORPH, 1-100;

charset Subset1 = 263674-263723 1-172 106679-106916 40313-40507 121157-121207 268519-268691 72785-72878 64031-64146;

charset Subset2 = 222957-223113 37699-37887 163777-163891 180308-180369 141676-142155 104509-104748 29946-30351 173-500;

charset Subset3 = 74718-74854 41925-42131 187821-188080 295033-295162 96131-96241 228451-228500 43196-43329 193559-193655 162713-162865 501-713;

charset Subset4 = 714-763 92964-93260 172402-172579 124352-124440;

charset Subset5 = 15940-16284 276605-277018 44544-44913 764-1171 255855-256239 265698-266248 93431-93899;

charset Subset6 = 1172-1296;

charset Subset7 = 174450-174750 299771-300054 78163-78369 292412-292575 1297-1393 257575-257776;

charset Subset8 = 1394-1730 46806-47305 276179-276462 230445-230973 188222-188922 231912-232155 233207-233812;

charset Subset9 = 1731-2062;

charset Subset10 = 2063-2112 62320-62369;

charset Subset11 = 236768-237202 76012-76252 23033-23163 64737-65411 2113-2497 208300-208564 232519-232828;

charset Subset12 = 87975-88335 69890-70140 2498-2926;

charset Subset13 = 2927-3106 82289-82548 120327-120499 73584-73708 102112-102237 82549-82676;

charset Subset14 = 249765-250117 3107-3242;

charset Subset15 = 100613-100682 3243-3294 16919-17019 299205-299303 235798-235852;

charset Subset16 = 267409-267497 3295-3368;

charset Subset17 = 221703-222055 172244-172350 177287-177792 67057-67295 3369-3941 241538-242008 142570-142994 217060-217182;

charset Subset18 = 202047-202098 3942-4062;

charset Subset19 = 219832-220242 126157-126450 4063-4494 176545-177041 258415-258771 301813-302408 6590-6915;

charset Subset20 = 222056-222171 4495-4618;

charset Subset21 = 4619-4670 96433-96616 135941-135990;

charset Subset22 = 27208-27304 27736-27818 241195-241484 79519-79840 256381-256506 4671-4894;

charset Subset23 = 256507-257061 189861-190002 4895-5256;

charset Subset24 = 19365-19492 5257-5463 287135-287297 141571-141675 301559-301722 263854-264133;

charset Subset25 = 5464-5513;

charset Subset26 = 25069-25143 5514-5563 68967-69036;

charset Subset27 = 5564-5867 219032-219193;

charset Subset28 = 5868-6101 266364-266620 250190-250500;

charset Subset29 = 6102-6389 130439-130549 197733-197831;

charset Subset30 = 6390-6439;

charset Subset31 = 247729-248046 6440-6589 255025-255109 295163-295306 37888-38226;

charset Subset32 = 195843-196024 236362-236485 6916-7004 208849-209023;

charset Subset33 = 8808-8893 133309-133399 235624-235797 7005-7131 18772-18862 144691-144961;

charset Subset34 = 7132-7550 26219-26429 119754-120326;

charset Subset35 = 7551-7733 286894-287134 223114-223265;

charset Subset36 = 7734-7970 171999-172243;

charset Subset37 = 218982-219031 7971-8022;

charset Subset38 = 8023-8112 272761-272948 239295-239346 44369-44543 48109-48165;

charset Subset39 = 152637-152788 213601-213796 24239-24295 279673-279736 141404-141570 246477-246594 56656-56775 8113-8162 151221-151347 264380-264519 92330-92428;

charset Subset40 = 8163-8474 222820-222956 80703-80894 225365-225668 72280-72784 97263-97617;

charset Subset41 = 74279-74330 8475-8698 86514-86646 295649-295793 101247-101398 89982-90122;

charset Subset42 = 100126-100313 8699-8807 261947-262030 151000-151064 190189-190324 89198-89312 199838-199888 122996-123050 155146-155274;

charset Subset43 = 209024-209290 158948-159192 69037-69292 186171-186245 8894-9094;

charset Subset44 = 296953-297605 9095-9382;

charset Subset45 = 44914-45141 9383-9476 249443-249545 99701-99750;

charset Subset46 = 63410-63598 293042-293288 20619-20668 138919-139059 83799-83981 162618-162712 64147-64443 9477-9604 180431-180483 24788-25068 296487-296753;

charset Subset47 = 80061-80496 120988-121156 9605-9762;

charset Subset48 = 140890-141030 9763-9974 144166-144369 170603-170746 263724-263803;

charset Subset49 = 9975-10024 58012-58213;

charset Subset50 = 94004-94128 10025-10189 183686-183916 193656-194034 103434-103689 199659-199837 142995-143148;

charset Subset51 = 115190-115449 285667-286183 13866-14460 10190-10701 136781-137080 279737-280229 114481-114889 52367-52846 66944-67056;

charset Subset52 = 44028-44368 126681-126798 82986-83165 10702-10756 126451-126628 158571-158672 276017-276178 258355-258414 159532-159648;

charset Subset53 = 97042-97262 10757-11096 131486-131539 152789-152906 11798-11952 52847-52969 177042-177091;

charset Subset54 = 11097-11283 109258-109476 259386-259695 57329-57465 57682-58011 288745-289104 186826-187069 268796-268974 23435-23552;

charset Subset55 = 11284-11699 70752-70862 198979-199160 264963-265069;

charset Subset56 = 237203-237272 48675-48743 11700-11797;

charset Subset57 = 245642-245954 88386-88977 66023-66252 253742-253843 103168-103433 11953-12614 235853-236299 17131-17343;

charset Subset58 = 232206-232518 12615-12905 67357-67439;

charset Subset59 = 95545-95860 65465-65662 165685-165880 12906-13477 77460-77729;

charset Subset60 = 13478-13532 125325-125375;

charset Subset61 = 112234-112562 122811-122995 13533-13725 185880-186006 35932-36410;

charset Subset62 = 129429-129510 234570-234816 19292-19364 13726-13865 221066-221169;

charset Subset63 = 284000-284348 126629-126680 14461-14776;

charset Subset64 = 14777-14909;

charset Subset65 = 283768-283999 98267-98443 166986-167432 160846-161281 75694-76011 280556-280769 298143-298380 115992-116074 259147-259385 186614-186774 14910-15100 163892-164255 93351-93430;

charset Subset66 = 15101-15728 62370-62523;

charset Subset67 = 90175-90714 85483-85795 31737-32285 159649-159994 15729-15790;

charset Subset68 = 82172-82288 15791-15874 68859-68966 241485-241537 283377-283536 61142-61261 117516-117700;

charset Subset69 = 121517-121647 184822-184871 15875-15939;

charset Subset70 = 227030-227167 69748-69819 84436-84486 16285-16399 268692-268795;

charset Subset71 = 198371-198461 66253-66302 28722-28828 215294-215410 16400-16540 21276-21543 101399-101450;

charset Subset72 = 16541-16918 100386-100612;

charset Subset73 = 67296-67356 223756-223855 155746-155947 150078-150196 145596-145856 52970-53195 17020-17130;

charset Subset74 = 17344-17836 79084-79518 260878-261252 227168-227686;

charset Subset75 = 61471-61603 26149-26218 200175-200288 169715-169902 17837-18048;

charset Subset76 = 262130-262548 189185-189386 252610-252846 18049-18277;

charset Subset77 = 216788-217059 47883-48108 166849-166985 113764-114077 259041-259146 154173-154373 18278-18348 42771-42981 50886-50951 218666-218842;

charset Subset78 = 18349-18512 266249-266363 33863-34049 246248-246476 84487-84636;

charset Subset79 = 174751-174995 268042-268518 257986-258354 261673-261946 123965-124117 252161-252425 18513-18714 292348-292411;

charset Subset80 = 18715-18771 251616-251719 111298-111518 190003-190188 229439-229577;

charset Subset81 = 245288-245641 223906-224295 293909-294107 18863-19291 152196-152636 90715-90928 110055-110628 290275-290524 115626-115941;

charset Subset82 = 199411-199658 75029-75339 19493-19710 228501-228580 250501-251077 278756-279006 139060-139696;

charset Subset83 = 19711-19764 264258-264379;

charset Subset84 = 19765-19816 191017-191116;

charset Subset85 = 115942-115991 19817-19881 64668-64736 58717-58806;

charset Subset86 = 19882-20142 234885-235623;

charset Subset87 = 53707-53835 20143-20329 110782-110941 206989-207235 166576-166772 91117-91202 146964-147063 138080-138234 117701-117818;

charset Subset88 = 167608-167961 20330-20473 272657-272760;

charset Subset89 = 20474-20618;

charset Subset90 = 20669-20830 102908-103167;

charset Subset91 = 233813-233906 210230-210364 20831-20888 274562-274910;

charset Subset92 = 20889-21223 201193-201376 96242-96432 173944-174449;

charset Subset93 = 93900-94003 280230-280555 195782-195842 21224-21275;

charset Subset94 = 104268-104508 21544-21777 254600-254885 55180-55550 75340-75693;

charset Subset95 = 246094-246247 21778-21923 257411-257574 244789-245077;

charset Subset96 = 199161-199410 278496-278755 21924-22181;

charset Subset97 = 22182-22530 229655-230181 197384-197630;

charset Subset98 = 24296-24578 283537-283767 186007-186170 22531-23032 229318-229438;

charset Subset99 = 23164-23434;

charset Subset100 = 69695-69747 238101-238171 134775-134964 23553-23681;

charset Subset101 = 257777-257985 97618-97825 281705-281859 55906-56090 168194-168403 140787-140839 228581-228759 23682-23961 109642-109706;

charset Subset102 = 96748-96991 23962-24152 82677-82985 199889-200174 91873-92329 93261-93350;

charset Subset103 = 29027-29150 24153-24238;

charset Subset104 = 70863-71001 173690-173787 115450-115625 300349-300486 43495-43607 178921-179164 24579-24707 142413-142569;

charset Subset105 = 24708-24787 186246-186328 141082-141131 248883-248932 274154-274206;

charset Subset106 = 25144-25384;

charset Subset107 = 25385-25515 171054-171826 99014-99528;

charset Subset108 = 25516-25678 171827-171876;

charset Subset109 = 274207-274561 77066-77361 25679-25928 272949-273451;

charset Subset110 = 58807-58954 225259-225364 271105-271189 41109-41335 159995-160201 25929-26018 102627-102727;

charset Subset111 = 155948-156026 26019-26148 176112-176274;

charset Subset112 = 233075-233206 26430-26585 30467-30561 160202-160258 192799-193076 105392-105459;

charset Subset113 = 26586-26728 83982-84031;

charset Subset114 = 289889-290133 237652-238100 206403-206988 42132-42440 254886-255024 26729-27093;

charset Subset115 = 269831-270017 27094-27207;

charset Subset116 = 108308-108610 61262-61470 95020-95313 27305-27735 191117-191458 285261-285476 128414-128586;

charset Subset117 = 260561-260624 27819-28184 52051-52366;

charset Subset118 = 30417-30466 28185-28306;

charset Subset119 = 28307-28384;

charset Subset120 = 28385-28552 292267-292347;

charset Subset121 = 217714-218201 28553-28721 249023-249442 151065-151220 224561-224831 147574-148107;

charset Subset122 = 218843-218981 28829-28895;

charset Subset123 = 211169-211413 37348-37698 28896-28972 118763-118919;

charset Subset124 = 28973-29026;

charset Subset125 = 29151-29575 43883-44027 122279-122810 34309-34665 219249-219570 91203-91629 121208-121516 56091-56655;

charset Subset126 = 78612-78952 242737-242841 29576-29744 243489-243631 299616-299770 45142-45219;

charset Subset127 = 170747-171053 263308-263587 71329-71389 149112-149178 29745-29945 48562-48674;

charset Subset128 = 30352-30416 119681-119753 57277-57328 196607-196677;

charset Subset129 = 41586-41924 48744-49174 264520-264912 30562-30976 254150-254599 62218-62319;

charset Subset130 = 197631-197682 133400-133675 30977-31157 275347-275551 148500-148821 123051-123155;

charset Subset131 = 177852-178136 156807-157215 31158-31685 209427-209809 196678-197223 140475-140786 295952-296486;

charset Subset132 = 73709-73941 159193-159253 31686-31736 74855-75028 83166-83334;

charset Subset133 = 32286-32463 141031-141081 45854-45977 179369-179537 113454-113763 278130-278495;

charset Subset134 = 32464-32513 244242-244430 113100-113269;

charset Subset135 = 224296-224415 32514-32676;

charset Subset136 = 228760-229109 32677-32937 202152-202271 195027-195353;

charset Subset137 = 125131-125324 215237-215293 32938-33242 175413-175477 180484-180536 288517-288744;

charset Subset138 = 81953-82171 209960-210229 284349-284770 33243-33862 241054-241194 292576-292947;

charset Subset139 = 151932-152195 185234-185385 154374-154527 34050-34113 67900-68189 65663-66022;

charset Subset140 = 205769-205865 242009-242088 128725-128990 261408-261672 51493-51762 39725-39857 34114-34308 104024-104267;

charset Subset141 = 34666-34961 253467-253661 252426-252523 127422-127754;

charset Subset142 = 207236-207342 270292-270596 140425-140474 205003-205195 216530-216787 34962-35286;

charset Subset143 = 271989-272539 35287-35553 271190-271636;

charset Subset144 = 35554-35746 107749-108051;

charset Subset145 = 80553-80702 161423-161518 35747-35931;

charset Subset146 = 218202-218319 165985-166271 132483-132752 36411-36501 40978-41108 91630-91776;

charset Subset147 = 219663-219831 290525-290901 36502-36818;

charset Subset148 = 36819-37018 236486-236644 211997-212427 159254-159395 42982-43195 205196-205768 70343-70751 40508-40977;

charset Subset149 = 87817-87974 104749-104872 70141-70342 37019-37347;

charset Subset150 = 38227-38985 105460-105820 201697-202046 164737-164871 138564-138855;

charset Subset151 = 261253-261407 226045-226118 238631-238766 38986-39062 194894-195026;

charset Subset152 = 298433-298721 202390-202524 203168-203697 301340-301558 39063-39113;

charset Subset153 = 39114-39163 294573-295032;

charset Subset154 = 98444-98815 39164-39673 258772-259040;

charset Subset155 = 39674-39724 66788-66943 156027-156162 187451-187605 61026-61141 185683-185879 224832-225024;

charset Subset156 = 251204-251615 238824-239294 39858-40260;

charset Subset157 = 40261-40312 247273-247384;

charset Subset158 = 41336-41585 116870-117515 137544-137866 214716-215236 289586-289888 120500-120987;

charset Subset159 = 89903-89981 42441-42770 149179-149435 230974-231130;

charset Subset160 = 45220-45341 49175-49273 43330-43494 87729-87816;

charset Subset161 = 135503-135875 55551-55647 255110-255736 43608-43814;

charset Subset162 = 131809-131898 43815-43882;

charset Subset163 = 45342-45853 163350-163776 84032-84435;

charset Subset164 = 45978-46334;

charset Subset165 = 49274-49673 46335-46696;

charset Subset166 = 217518-217713 111684-111733 46697-46805;

charset Subset167 = 47306-47357;

charset Subset168 = 301154-301339 47358-47447 300487-300540;

charset Subset169 = 194398-194722 47448-47882 300541-301056 185386-185682 187606-187820 257062-257113 58955-59552;

charset Subset170 = 48166-48561 81084-81154;

charset Subset171 = 49674-49836 107570-107748 169621-169714 225750-225985;

charset Subset172 = 49837-50093;

charset Subset173 = 50094-50187 293674-293748 131899-131959 250118-250189;

charset Subset174 = 215929-216004 178137-178290 50188-50335 65412-65464;

charset Subset175 = 50336-50885 162866-163170;

charset Subset176 = 150407-150999 217350-217517 265070-265423 50952-51411 196025-196340 148282-148499 56776-57024;

charset Subset177 = 185081-185233 277019-277175 95861-96130 189387-189436 51412-51492 122022-122175;

charset Subset178 = 215616-215928 282492-282639 51763-52000 162467-162617;

charset Subset179 = 98816-99013 52001-52050;

charset Subset180 = 53196-53435 291623-292266 86120-86513 183267-183685;

charset Subset181 = 130695-130821 139697-139799 124735-125011 57123-57276 53436-53706 77730-78162 285477-285589 262549-262720;

charset Subset182 = 204411-204885 148822-149111 228099-228450 74331-74717 53836-54219;

charset Subset183 = 87291-87366 54220-54333 296875-296952;

charset Subset184 = 102728-102907 245078-245220 146063-146349 96992-97041 54334-54704 284937-285260 293749-293908;

charset Subset185 = 234262-234569 84637-84778 299304-299615 54705-54968;

charset Subset186 = 54969-55179 136429-136673 286184-286346 60378-60729;

charset Subset187 = 221334-221650 137867-138079 55648-55905 233907-234261;

charset Subset188 = 57025-57122;

charset Subset189 = 57466-57592 279451-279672 264913-264962;

charset Subset190 = 104873-104985 194723-194893 274104-274153 57593-57681 236300-236361 95314-95411;

charset Subset191 = 118159-118275 242932-243276 301057-301153 58214-58360;

charset Subset192 = 193155-193558 58361-58716 222323-222744 243277-243488;

charset Subset193 = 245955-246093 239847-240064 128349-128413 231691-231911 59553-59686 106417-106678;

charset Subset194 = 232156-232205 59687-59779;

charset Subset195 = 179165-179368 202675-203048 59780-60221 126799-127421;

charset Subset196 = 135876-135940 67440-67578 191459-191556 60222-60377;

charset Subset197 = 149436-149646 239531-239846 60730-61025 264134-264257;

charset Subset198 = 290902-291475 61604-62130;

charset Subset199 = 157216-157265 192039-192243 62131-62217;

charset Subset200 = 62524-62863 240115-240674 101551-102052;

charset Subset201 = 62864-63293 84779-85071;

charset Subset202 = 63294-63409 78953-79083 301723-301812 63599-63689;

charset Subset203 = 281089-281704 111519-111683 83335-83798 300055-300348 63690-64030;

charset Subset204 = 212962-213158 64444-64667;

charset Subset205 = 66303-66787;

charset Subset206 = 134530-134774 67579-67899 170064-170237 130822-131200;

charset Subset207 = 295307-295648 287853-288392 68190-68858;

charset Subset208 = 69293-69694 119602-119680 166773-166848;

charset Subset209 = 145356-145595 163171-163349 69820-69889 140375-140424;

charset Subset210 = 178800-178920 71002-71146 118455-118762 97876-98188 164443-164686;

charset Subset211 = 111734-111964 71147-71328 158521-158570;

charset Subset212 = 71390-71931 133676-134144 218320-218665 270018-270291;

charset Subset213 = 71932-72162 279211-279450;

charset Subset214 = 160783-160845 162417-162466 72163-72279 81155-81330 198462-198583;

charset Subset215 = 109707-109918 143149-143352 72879-73152 213544-213600 146511-146963;

charset Subset216 = 277859-278129 87474-87728 161282-161422 220399-220908 297606-298142 128991-129428 73153-73583 121809-122021;

charset Subset217 = 127755-128052 73942-74193 271769-271923;

charset Subset218 = 89313-89417 74194-74278 279007-279160;

charset Subset219 = 76253-76556 77362-77459 203049-203167 176395-176544 237540-237651 227795-228098;

charset Subset220 = 76557-77015;

charset Subset221 = 77016-77065;

charset Subset222 = 78370-78611 246595-247272 180537-181342 114078-114332 124441-124635 191831-192038;

charset Subset223 = 284771-284936 168643-168919 90929-90992 79841-80060;

charset Subset224 = 80497-80552 128587-128724 101451-101550;

charset Subset225 = 80895-81083;

charset Subset226 = 81331-81952 156465-156755 155275-155745 226844-226979 198143-198370;

charset Subset227 = 85072-85230 291476-291525 182677-182880;

charset Subset228 = 280770-280908 85231-85482;

charset Subset229 = 273452-273501 85796-85852;

charset Subset230 = 210365-210696 273502-273556 86947-87290 85853-85925 175478-175830;

charset Subset231 = 110942-111297 85926-85982 135453-135502 197832-197881;

charset Subset232 = 265477-265697 210697-210819 239347-239530 85983-86119;

charset Subset233 = 223856-223905 206116-206402 202099-202151 86647-86946;

charset Subset234 = 150197-150406 271036-271104 275249-275346 87367-87473 192244-192511 186775-186825;

charset Subset235 = 88336-88385 139800-139956 121648-121808 219194-219248;

charset Subset236 = 207805-207997 182881-182965 125949-126156 194163-194218 88978-89197;

charset Subset237 = 172631-173052 198584-198978 225025-225258 89418-89902 273820-274103;

charset Subset238 = 90123-90174 197224-197276;

charset Subset239 = 277721-277858 148108-148281 252050-252160 90993-91116 137331-137543 114890-115139 211899-211996 223266-223317 135991-136172;

charset Subset240 = 91777-91872;

charset Subset241 = 92429-92963;

charset Subset242 = 181517-181971 94129-94819 138235-138366 223318-223755;

charset Subset243 = 94820-94931;

charset Subset244 = 94932-95019 170440-170602;

charset Subset245 = 201427-201696 191557-191830 289333-289585 238767-238823 294108-294265 95412-95544;

charset Subset246 = 272540-272656 189437-189860 273557-273819 202272-202389 96617-96747;

charset Subset247 = 97826-97875;

charset Subset248 = 276463-276548 98189-98266 271637-271768;

charset Subset249 = 99529-99633 253012-253079;

charset Subset250 = 99634-99700;

charset Subset251 = 299048-299204 99751-100125;

charset Subset252 = 107161-107222 216005-216267 259696-259927 100314-100385 287665-287802;

charset Subset253 = 170238-170439 100683-100732 219571-219662;

charset Subset254 = 281915-282207 100733-100838 158673-158947 195500-195781 184872-185080;

charset Subset255 = 100839-100891;

charset Subset256 = 102238-102626 100892-101246 123156-123736;

charset Subset257 = 102053-102111 124118-124351 189003-189184 270597-270891 132285-132482 255737-255854 227687-227794 196341-196606 205866-205984 165881-165984 249546-249764;

charset Subset258 = 243863-244241 103690-104023 274961-275248 190325-190730;

charset Subset259 = 104986-105051 282208-282429;

charset Subset260 = 216268-216529 270892-271035 105052-105391 253080-253466 131540-131808;

charset Subset261 = 105821-106131;

charset Subset262 = 106132-106211;

charset Subset263 = 144962-145355 203911-204139 146350-146510 164922-165502 106212-106416;

charset Subset264 = 116242-116336 106917-107160 168972-169198 207674-207804 251770-251990;

charset Subset265 = 107223-107569;

charset Subset266 = 134965-135160 108052-108307 193077-193154;

charset Subset267 = 108611-108716 230240-230444 226560-226788 211031-211168 202525-202674;

charset Subset268 = 108717-108940;

charset Subset269 = 173341-173585 136173-136428 149894-150077 108941-109160 259928-260560 145857-146062;

charset Subset270 = 281860-281914 109161-109257;

charset Subset271 = 109477-109641 128053-128348 118276-118454 220243-220398;

charset Subset272 = 109919-110054 212428-212682 257114-257410 200289-200410;

charset Subset273 = 153764-154172 110629-110781 175292-175412;

charset Subset274 = 298722-299047 111965-112233;

charset Subset275 = 124636-124734 112563-112685 289105-289332;

charset Subset276 = 177092-177286 141132-141403 112686-112979;

charset Subset277 = 180370-180430 112980-113099;

charset Subset278 = 231219-231538 113270-113453 152959-153665;

charset Subset279 = 231131-231218 114333-114412;

charset Subset280 = 221651-221702 220968-221065 267834-267918 214582-214715 197882-198142 114413-114480;

charset Subset281 = 240065-240114 115140-115189;

charset Subset282 = 282640-282919 116075-116241 188923-189002;

charset Subset283 = 116337-116796;

charset Subset284 = 212683-212961 116797-116869 251078-251134;

charset Subset285 = 242842-242931 117819-118158 152907-152958;

charset Subset286 = 209291-209359 118920-118977;

charset Subset287 = 118978-119275;

charset Subset288 = 119276-119601;

charset Subset289 = 240871-241053 149728-149893 122176-122278;

charset Subset290 = 167433-167607 123737-123964 143806-143861;

charset Subset291 = 263588-263673 179538-180121 125012-125130 166272-166575 267919-268041;

charset Subset292 = 125376-125895;

charset Subset293 = 125896-125948 147408-147573;

charset Subset294 = 207343-207673 129511-129689 181972-182116;

charset Subset295 = 157467-158116 129690-130264;

charset Subset296 = 173586-173689 229578-229654 130265-130388 194219-194397 271924-271988;

charset Subset297 = 130389-130438;

charset Subset298 = 130550-130694;

charset Subset299 = 131201-131485;

charset Subset300 = 213797-214275 131960-132284;

charset Subset301 = 275552-275801 226119-226559 132753-133308 192512-192798;

charset Subset302 = 134145-134198 183917-184214;

charset Subset303 = 142156-142412 134199-134529 177793-177851;

charset Subset304 = 135161-135452;

charset Subset305 = 136674-136780;

charset Subset306 = 178291-178424 137081-137330 285590-285666 298381-298432 176275-176394 138856-138918 224416-224560 256240-256380 295794-295951;

charset Subset307 = 215411-215615 138367-138513;

charset Subset308 = 251135-251203 169199-169345 248933-249022 138514-138563;

charset Subset309 = 139957-140374 293289-293673 160259-160782;

charset Subset310 = 200853-201142 247679-247728 140840-140889;

charset Subset311 = 143353-143805;

charset Subset312 = 143862-144165 238172-238244;

charset Subset313 = 154591-155145 144370-144690;

charset Subset314 = 147064-147251;

charset Subset315 = 244431-244788 147252-147407 267498-267833;

charset Subset316 = 209810-209959 149647-149727;

charset Subset317 = 151348-151931 168404-168642 205985-206115;

charset Subset318 = 220909-220967 287803-287852 153666-153763;

charset Subset319 = 154528-154590 279161-279210;

charset Subset320 = 253844-254085 158171-158520 156163-156464;

charset Subset321 = 159396-159531 156756-156806 165503-165684 236645-236767;

charset Subset322 = 157266-157466 182966-183266;

charset Subset323 = 248541-248882 158117-158170 237273-237539;

charset Subset324 = 161519-162024 248047-248241;

charset Subset325 = 162025-162176;

charset Subset326 = 162177-162416 231539-231690;

charset Subset327 = 164256-164442;

charset Subset328 = 201143-201192 164687-164736;

charset Subset329 = 164872-164921;

charset Subset330 = 232829-233074 167962-168193;

charset Subset331 = 168920-168971;

charset Subset332 = 190731-191016 204345-204410 169346-169620 204140-204344;

charset Subset333 = 169903-170063;

charset Subset334 = 171877-171998 296754-296874 172351-172401 263804-263853;

charset Subset335 = 172580-172630;

charset Subset336 = 173053-173340;

charset Subset337 = 265424-265476 173788-173943 253662-253741;

charset Subset338 = 174996-175291;

charset Subset339 = 175831-176111 287298-287664;

charset Subset340 = 195354-195443 276549-276604 178425-178799;

charset Subset341 = 180122-180307;

charset Subset342 = 181343-181516 248242-248328;

charset Subset343 = 277249-277559 182117-182676 238245-238630;

charset Subset344 = 184215-184546 186329-186613 242089-242736;

charset Subset345 = 184547-184821;

charset Subset346 = 187070-187450 260684-260877;

charset Subset347 = 188081-188221 280964-281088;

charset Subset348 = 221170-221333 194035-194162;

charset Subset349 = 274911-274960 195444-195499 226789-226843;

charset Subset350 = 197277-197383;

charset Subset351 = 234817-234884 197683-197732;

charset Subset352 = 200411-200852;

charset Subset353 = 201377-201426;

charset Subset354 = 207998-208299 260625-260683 203698-203845;

charset Subset355 = 214276-214376 222745-222819 203846-203910;

charset Subset356 = 204886-205002;

charset Subset357 = 208565-208848;

charset Subset358 = 291526-291622 209360-209426 222172-222322;

charset Subset359 = 286500-286893 210820-211030 214377-214581;

charset Subset360 = 211414-211688;

charset Subset361 = 211689-211898;

charset Subset362 = 213159-213543 283119-283320;

charset Subset363 = 217183-217349;

charset Subset364 = 225669-225749;

charset Subset365 = 225986-226044;

charset Subset366 = 226980-227029;

charset Subset367 = 230182-230239 229110-229317;

charset Subset368 = 288393-288516 245221-245287 243632-243862 262031-262129 240675-240870;

charset Subset369 = 248329-248540 247385-247528;

charset Subset370 = 267216-267408 247529-247678;

charset Subset371 = 251720-251769;

charset Subset372 = 251991-252049;

charset Subset373 = 252524-252609;

charset Subset374 = 252847-253011 302409-302543 294266-294572;

charset Subset375 = 254086-254149;

charset Subset376 = 269080-269830 262721-263307;

charset Subset377 = 266621-266890;

charset Subset378 = 266891-267149;

charset Subset379 = 267150-267215;

charset Subset380 = 268975-269079;

charset Subset381 = 275802-276016;

charset Subset382 = 277176-277248;

charset Subset383 = 277560-277720 292948-293041;

charset Subset384 = 280909-280963;

charset Subset385 = 282430-282491;

charset Subset386 = 282920-283118;

charset Subset387 = 283321-283376;

charset Subset388 = 286347-286499;

charset Subset389 = 290134-290274;

charset Subset390 = 302544-333766;

charpartition PartitionFinder = MK+ASC:Morph, GTR+G:Subset1, GTR+I+G:Subset2, GTR+G:Subset3, GTR+G:Subset4, GTR+I+G:Subset5, GTR+G:Subset6, GTR+G:Subset7, GTR+I+G:Subset8, GTR+G:Subset9, GTR+I+G:Subset10, GTR+I+G:Subset11, GTR+I+G:Subset12, GTR+G:Subset13, GTR+I+G:Subset14, GTR+G:Subset15, GTR+G:Subset16, GTR+I+G:Subset17, GTR+G:Subset18, GTR+I+G:Subset19, GTR+G:Subset20, GTR+G:Subset21, GTR+G:Subset22, GTR+I+G:Subset23, GTR+I+G:Subset24, GTR+G:Subset25, GTR+G:Subset26, GTR+I+G:Subset27, GTR+G:Subset28, GTR+I+G:Subset29, GTR+G:Subset30, GTR+G:Subset31, GTR+G:Subset32, GTR+G:Subset33, GTR+I+G:Subset34, GTR+G:Subset35, GTR+I+G:Subset36, GTR+G:Subset37, GTR+G:Subset38, GTR+G:Subset39, GTR+I+G:Subset40, GTR+G:Subset41, GTR+G:Subset42, GTR+G:Subset43, GTR+I+G:Subset44, GTR+I+G:Subset45, GTR+G:Subset46, GTR+G:Subset47, GTR+G:Subset48, GTR+G:Subset49, GTR+G:Subset50, GTR+I+G:Subset51, GTR+G:Subset52, GTR+I+G:Subset53, GTR+I+G:Subset54, GTR+G:Subset55, GTR+I+G:Subset56, GTR+G:Subset57, GTR+G:Subset58, GTR+I+G:Subset59, GTR+G:Subset60, GTR+G:Subset61, GTR+G:Subset62, GTR+G:Subset63, GTR+I+G:Subset64, GTR+G:Subset65, GTR+I+G:Subset66, GTR+I+G:Subset67, GTR+G:Subset68, GTR+G:Subset69, GTR+G:Subset70, GTR+G:Subset71, GTR+I+G:Subset72, GTR+G:Subset73, GTR+I+G:Subset74, GTR+G:Subset75, GTR+G:Subset76, GTR+G:Subset77, GTR+G:Subset78, GTR+G:Subset79, GTR+I+G:Subset80, GTR+I+G:Subset81, GTR+I+G:Subset82, GTR+G:Subset83, GTR+G:Subset84, GTR+G:Subset85, GTR+G:Subset86, GTR+G:Subset87, GTR+I+G:Subset88, GTR+G:Subset89, GTR+G:Subset90, GTR+G:Subset91, GTR+I+G:Subset92, GTR+G:Subset93, GTR+G:Subset94, GTR+G:Subset95, GTR+G:Subset96, GTR+G:Subset97, GTR+I+G:Subset98, GTR+G:Subset99, GTR+G:Subset100, GTR+G:Subset101, GTR+I+G:Subset102, GTR+G:Subset103, GTR+G:Subset104, GTR+I+G:Subset105, GTR+I+G:Subset106, GTR+I+G:Subset107, GTR+G:Subset108, GTR+I+G:Subset109, GTR+G:Subset110, GTR+G:Subset111, GTR+G:Subset112, GTR+I+G:Subset113, GTR+I+G:Subset114, GTR+I+G:Subset115, GTR+I+G:Subset116, GTR+I+G:Subset117, GTR+G:Subset118, GTR+I+G:Subset119, GTR+G:Subset120, GTR+I+G:Subset121, GTR+I+G:Subset122, GTR+G:Subset123, GTR+G:Subset124, GTR+I+G:Subset125, GTR+G:Subset126, GTR+G:Subset127, GTR+G:Subset128, GTR+I+G:Subset129, GTR+G:Subset130, GTR+I+G:Subset131, GTR+G:Subset132, GTR+G:Subset133, GTR+G:Subset134, GTR+G:Subset135, GTR+I+G:Subset136, GTR+G:Subset137, GTR+I+G:Subset138, GTR+G:Subset139, GTR+G:Subset140, GTR+G:Subset141, GTR+G:Subset142, GTR+G:Subset143, GTR+G:Subset144, GTR+G:Subset145, GTR+G:Subset146, GTR+G:Subset147, GTR+G:Subset148, GTR+G:Subset149, GTR+I+G:Subset150, GTR+G:Subset151, GTR+G:Subset152, GTR+G:Subset153, GTR+I+G:Subset154, GTR+G:Subset155, GTR+G:Subset156, GTR+G:Subset157, GTR+I+G:Subset158, GTR+G:Subset159, GTR+G:Subset160, GTR+I+G:Subset161, GTR+G:Subset162, GTR+I+G:Subset163, GTR+I+G:Subset164, GTR+G:Subset165, GTR+G:Subset166, GTR+G:Subset167, GTR:Subset168, GTR+I+G:Subset169, GTR+I+G:Subset170, GTR+I+G:Subset171, GTR+G:Subset172, GTR+G:Subset173, GTR+G:Subset174, GTR+I+G:Subset175, GTR+G:Subset176, GTR+G:Subset177, GTR+I+G:Subset178, GTR+G:Subset179, GTR+I+G:Subset180, GTR+G:Subset181, GTR+I+G:Subset182, GTR+G:Subset183, GTR+G:Subset184, GTR+G:Subset185, GTR+G:Subset186, GTR+G:Subset187, GTR+G:Subset188, GTR+G:Subset189, GTR+G:Subset190, GTR+G:Subset191, GTR+I+G:Subset192, GTR+G:Subset193, GTR+G:Subset194, GTR+I+G:Subset195, GTR+G:Subset196, GTR+G:Subset197, GTR+I+G:Subset198, GTR+G:Subset199, GTR+I+G:Subset200, GTR+G:Subset201, GTR+G:Subset202, GTR+I+G:Subset203, GTR+G:Subset204, GTR+G:Subset205, GTR+I+G:Subset206, GTR+G:Subset207, GTR+G:Subset208, GTR+G:Subset209, GTR+I+G:Subset210, GTR+G:Subset211, GTR+G:Subset212, GTR+G:Subset213, GTR+G:Subset214, GTR+G:Subset215, GTR+I+G:Subset216, GTR+G:Subset217, GTR+G:Subset218, GTR+G:Subset219, GTR+I+G:Subset220, GTR+G:Subset221, GTR+I+G:Subset222, GTR+G:Subset223, GTR+G:Subset224, GTR+G:Subset225, GTR+I+G:Subset226, GTR+G:Subset227, GTR+I+G:Subset228, GTR+I+G:Subset229, GTR+I+G:Subset230, GTR+I+G:Subset231, GTR+G:Subset232, GTR+G:Subset233, GTR+G:Subset234, GTR+G:Subset235, GTR+G:Subset236, GTR+I+G:Subset237, GTR+G:Subset238, GTR+G:Subset239, GTR+I+G:Subset240, GTR+I+G:Subset241, GTR+I+G:Subset242, GTR+G:Subset243, GTR+G:Subset244, GTR+G:Subset245, GTR+G:Subset246, GTR+G:Subset247, GTR+G:Subset248, GTR+G:Subset249, GTR+I+G:Subset250, GTR+G:Subset251, GTR+I+G:Subset252, GTR+G:Subset253, GTR+I+G:Subset254, GTR+G:Subset255, GTR+G:Subset256, GTR+G:Subset257, GTR+I+G:Subset258, GTR+G:Subset259, GTR+G:Subset260, GTR+G:Subset261, GTR+G:Subset262, GTR+G:Subset263, GTR+I+G:Subset264, GTR+I+G:Subset265, GTR+G:Subset266, GTR+G:Subset267, GTR+G:Subset268, GTR+I+G:Subset269, GTR+G:Subset270, GTR+G:Subset271, GTR+G:Subset272, GTR+I+G:Subset273, GTR+I+G:Subset274, GTR+G:Subset275, GTR+I+G:Subset276, GTR+G:Subset277, GTR+G:Subset278, GTR+G:Subset279, GTR+G:Subset280, GTR+G:Subset281, GTR+G:Subset282, GTR+I+G:Subset283, GTR+G:Subset284, GTR+G:Subset285, GTR+G:Subset286, GTR+G:Subset287, GTR+G:Subset288, GTR+G:Subset289, GTR+G:Subset290, GTR+I+G:Subset291, GTR+I+G:Subset292, GTR+G:Subset293, GTR+G:Subset294, GTR+G:Subset295, GTR+G:Subset296, GTR+G:Subset297, GTR+G:Subset298, GTR+G:Subset299, GTR+I+G:Subset300, GTR+I+G:Subset301, GTR+G:Subset302, GTR+I+G:Subset303, GTR:Subset304, GTR+G:Subset305, GTR+G:Subset306, GTR+G:Subset307, GTR+G:Subset308, GTR+I+G:Subset309, GTR+G:Subset310, GTR+I+G:Subset311, GTR+G:Subset312, GTR+G:Subset313, GTR+G:Subset314, GTR+I+G:Subset315, GTR+G:Subset316, GTR+I+G:Subset317, GTR+G:Subset318, GTR+G:Subset319, GTR+I+G:Subset320, GTR+I+G:Subset321, GTR+G:Subset322, GTR+I+G:Subset323, GTR+G:Subset324, GTR+I+G:Subset325, GTR+G:Subset326, GTR+G:Subset327, GTR+G:Subset328, GTR+G:Subset329, GTR+G:Subset330, GTR+G:Subset331, GTR+G:Subset332, GTR+G:Subset333, GTR+I+G:Subset334, GTR+G:Subset335, GTR+I+G:Subset336, GTR+G:Subset337, GTR+G:Subset338, GTR+I+G:Subset339, GTR+G:Subset340, GTR+G:Subset341, GTR+G:Subset342, GTR+I+G:Subset343, GTR+G:Subset344, GTR+G:Subset345, GTR+G:Subset346, GTR+G:Subset347, GTR:Subset348, GTR+G:Subset349, GTR+G:Subset350, GTR+I+G:Subset351, GTR+G:Subset352, GTR+G:Subset353, GTR+I+G:Subset354, GTR+G:Subset355, GTR+G:Subset356, GTR+I+G:Subset357, GTR+G:Subset358, GTR+I+G:Subset359, GTR+G:Subset360, GTR+G:Subset361, GTR+I+G:Subset362, GTR+G:Subset363, GTR+G:Subset364, GTR+G:Subset365, GTR+I+G:Subset366, GTR+G:Subset367, GTR+G:Subset368, GTR+G:Subset369, GTR+G:Subset370, GTR+G:Subset371, GTR+G:Subset372, GTR+G:Subset373, GTR+G:Subset374, GTR:Subset375, GTR+I+G:Subset376, GTR+I+G:Subset377, GTR+I+G:Subset378, GTR+G:Subset379, GTR+G:Subset380, GTR+G:Subset381, GTR+G:Subset382, GTR+G:Subset383, GTR+G:Subset384, GTR:Subset385, GTR+G:Subset386, GTR:Subset387, GTR+G:Subset388, GTR+G:Subset389, GTR+I+G:Subset390;

end;
